# Social-Ecological Correlates of Children’s Independent Mobility: A Systematic Review

**DOI:** 10.3390/ijerph19031604

**Published:** 2022-01-30

**Authors:** Negin A. Riazi, Kelly Wunderlich, Lira Yun, Derek C. Paterson, Guy Faulkner

**Affiliations:** 1 Department of Health Sciences, Brock University, St. Catharines, ON L2S 3A1, Canada; 2 School of Kinesiology, The University of British Columbia, Vancouver, BC V6T 1Z4, Canada; kelly.wunderlich@ubc.ca (K.W.); Lira.Yun@albertahealthservices.ca (L.Y.); d.paterson@ubc.ca (D.C.P.); guy.faulkner@ubc.ca (G.F.)

**Keywords:** review, active travel, youth, public health, childhood, physical activity

## Abstract

Children’s independent mobility (IM) is associated with a range of benefits and understanding the factors that influence IM can support creation of effective interventions. The review (Prospero CRD42016042174) systematically summarized the available literature for social-ecological correlates of children’s IM in school-aged children and youth (aged 5–17 years). In this case, 53 studies were included and evaluated six individual, 15 interpersonal, 12 social environment, and 19 built environment- level variables. Most studies originated from Australia (*n* = 15) and Canada (*n* = 8) with most published in 2011 or later (*n* = 48). Variables that were consistently (positively and/or negatively) associated with children’s IM were age, ethnicity/race, child’s perceived competence, ownership of a house/access to house keys, having siblings, parents’ attitude toward IM and perception of child’s confidence, children’s interest in environment and activities, parents’ concern around traffic, housing/residential density, length of residency in one’s home, distance to destinations, and proximity to green space. Given the inter-related social-ecological correlates identified, intervention to promote children’s IM will likely need a multi-level and multi-sectoral approach. However, focus areas of building children’s skills and confidence, helping parents gain confidence in their children’s abilities, assuaging parental traffic concerns, and building environments with shorter distances to destinations of interest for children should be prioritized.

## 1. Introduction

Physical activity (PA) is associated with a number of benefits including improved cognitive functioning, physical, psychological, and social health [1,2]. However, globally most children and youth are not achieving PA recommendations requiring children and youth (5–17 years of age) to accumulate 60 min of moderate-to-vigorous PA daily [3]. Of the countries participating in The Global Matrix 3.0 the majority scored in the ‘D’ range for ‘overall PA’ on an ‘A’ to ‘F’ scale [4]. With growing concerns over the prevalence of physical inactivity in children and adolescents and the implications of childhood PA tracking into adulthood and positively influencing adult health outcomes [5,6], it is important to examine facilitators of PA during childhood.

While facilitators of PA may include access to sports participation or involvement in physical education [7], there is a growing interest in the role of children’s independent mobility (IM). Children’s IM refers to a child’s “freedom to travel around their own neighbourhood or city without adult supervision” [8] (p. 265) and it may play a vital role in helping children and youth achieve recommended levels of PA. Children’s IM has been operationalized in a number of ways within the literature, such as through IM licenses (e.g., crossing major roads, walking to school) reflecting the amount of freedom children are granted [9,10]. Children’s IM has also been measured via territorial range (e.g., how far from home can a child travel) [11], play participation (e.g., autonomous outdoor play), time of day (e.g., independent activity during the afterschool period) [12], and weekday versus weekend IM [13].

A systematic review of 52 studies by Schoeppe and colleagues (2013) found that children with the freedom to travel actively or play outdoors independently tend to accumulate more PA than their less independently mobile counterparts [14]. A study by Page and colleagues (2010) found that IM was related to increased likelihood of boys’ participation in play, structured sports and exercise, active commuting and girls’ active commuting [15]. The benefits of children’s IM move beyond the accumulation of PA and may also provide children with social, cognitive, and personal development benefits [12,16]. Children engaging in more freedom of movement within their environment tend to have a more comprehensive environmental knowledge of the context in which their home-to-school route is situated [11].

Over recent decades, there has been a general decline in levels of children’s IM. One landmark study by Hillman and colleagues found that children’s IM levels in England fell dramatically over the span of two decades, from 80% of 7–8-year-olds traveling independently to school in 1971, to only 9% in 1990 [9]. Similar trends have been seen in Germany [17] and Scandinavian countries such as Denmark, Finland and Norway [18]. These declines are attributed to a number of factors including increased car use, perceived dangers from traffic and ‘stranger danger’ [9,17,19], and what some argue is a risk-averse society [20]. These declining levels are concerning, especially as children’s IM may be an important facilitator of PA as well as providing children with cognitive and psychosocial development through peer interactions, development of wayfinding skills, risk assessment and enhancement of their decision-making skills [8]. It is therefore important to identify the factors which influence IM to inform research, policy and practice.

At the time of registering this review on PROSPERO (2016), no reviews of correlates of children’s IM existed. A few reviews have now been conducted with a focus on homes and communities [21], social and physical environments [22], or the built environment [23]. These reviews have highlighted the importance of the social environment for IM [22] with one review proposing the social environment may be an important mediator between environmental correlates and parents’ decision-making for IM [21]. A meta analytic review examining built environmental factors and IM found land use patterns and street design patterns to be significantly associated with IM; additionally, the review underscored the large amount of diversity amongst primary studies arising from differences in study locations, design, and characteristics of the included samples [23].

These reviews have highlighted the diversity of correlates influencing children’s IM. With the acceleration of studies examining IM, it is vital to provide an up-to-date synthesis of the broad range of factors influencing IM that can be used by multiple key stakeholders (e.g., city planners, parents, school boards). The purpose of this review is to synthesize and summarize the literature on the correlates of IM through a social-ecological lens. A social-ecological framework acknowledges that a health behaviour is impacted by multiple levels of influence and these influences on behaviour interact across different levels [24,25]. These different layers of influence may include factors at the individual level (e.g., children’s characteristics and behaviour), interpersonal level (e.g., household and parent characteristics), social-environment level (e.g., child and parent perceptions on traffic, stranger danger), built-environment level (e.g., density, design), policy level (e.g., school policies to promote active transportation), and natural-environment level (e.g., weather). Identifying the correlates of children’s IM may help inform future interventions, policies, and the development of effective strategies to increase children’s IM.

## 2. Methods

### 2.1. Search Strategy

The PRISMA guidelines [26] were utilized and the protocol was developed a priori and registered on PROSPERO (registration number: CRD42016042174). An internet search was conducted utilizing the following databases: PubMed (1946-present), Web of Science Core Collection (1900-present), PsycINFO (1880-present), EMBASE (1974-present), Sport Discus (1837-present), CINAHL (1982-present), Urban Studies Abstracts (1973-present). A department-specific certified librarian was consulted regarding the search strategy including databases and keywords. Keywords used to search for children’s IM articles included: “independent mobility” OR unsupervised OR “outdoor autonomy” OR self-reliance OR journey OR “outdoor play” OR excursion OR “active travel” OR “active transport *” OR active commut * OR “active play” AND child * OR youth * OR adolescen *.

### 2.2. Inclusion and Exclusion Criteria

The criteria for inclusion of studies in this review were as follows: (1) studies examined IM as the primary outcome variable; (2) studies examined correlates of IM; (3) studies focused on children and youth as the target sample of IM (i.e., school-aged children, 5–17 years); (4) studies had an observational design (longitudinal or cross-sectional); (5) studies were peer-reviewed and published; (6) studies were written in an English language. The search was not limited by publication date. The main exclusion criteria were: (1) studies of qualitative design as no outcome variables or statistical results were reported; (2) studies where IM was not the focus or IM was considered an independent variable; (3) the study was a protocol paper, review, meta-study, book chapter, conference proceeding or abstract.

### 2.3. Selection Process and Coding Associations

A web-based software program, EPPI-Reviewer 4 (Thomas et al., 2010), was used to manage and sort the studies extracted for this review. EPPI-Reviewer 4 was used for each stage of the review process including: reference management, screening, coding, and synthesis. Initial searches were run, and articles gathered from July to August 2017 by first author (NAR). A second search was run from the end of the first search until January 2019 (August 2017 to January 2019) and a final search was conducted from end of first search to October 2020 (January 2019–October 2020) as a significant amount of time had passed since initial searches were conducted. Duplicates were removed and title and abstract screening of the articles was performed by two independent reviewers (NAR (primary reviewer) and KW (1st search); LY (2nd search); DCP (final search)) to identify studies that met the inclusion criteria. Any disagreements were discussed between the reviewers and if needed, resolved via arbitration with a third reviewer (GF). Full text studies were screened, cross-checked, and data were extracted independently by two independent reviewers. Disagreements that arose were discussed amongst the two reviewers (NAR and KW, LY, or DCP) and were resolved through consensus with a third reviewer (GF). Additionally, the references of included studies were scanned for additional studies. Data from included studies were extracted including origin of study data, sample population, results, and correlates of IM.

### 2.4. Quality Assessment

The methodological quality of the studies was assessed using a 17-point criteria adapted from Schoeppe and colleagues (2013) for reporting on observational studies [14]. The modified checklist captured the quality of reporting as well as characteristics of the study quality. The quality of the studies was appraised independently by two reviewers (NAR and KW) and disagreements were resolved through consultation with another reviewer (LY). Each criterion was rated on a scale of 0 to 1 (0 = no/unclear; 0.5 = partial; 1 = yes) for fulfillment of the criterion. The highest score attainable for a study was 17; the score for each study was divided by the highest score possible and multiplied by 100 to provide an overall percentage of study quality. The studies were then grouped to represent high (>66.7%), fair (50–66.6%), or low (<50%) study quality [14].

### 2.5. Coding Associations and Classifying Variables

Studies included a wide range of statistical techniques to evaluate associations including correlations, *t*-tests, ANOVAs, and multivariate analyses (e.g., linear regression, logistic regression, structural equation modeling). The column ‘Related to children’s independent mobility’ indicates which studies reported significant associations (+ or −) between the variable and children’s IM, while the column ‘Unrelated to children’s independent mobility’ indicates studies with nonsignificant associations between the variable and children’s IM. There was variability in reporting between univariate and multivariate tests; if multivariate tests were conducted, the results from those tests were reported. Variables are not shown in summary tables unless three or more comparisons were found (see [27]). Some variables were combined if conceptually similar and there were not enough studies to examine the variables individually. For example, a ‘perceived competence’ category was created to encompass children’s perceptions of their safety experience, maturity, and confidence related to IM. The summary column represents the percentage found by taking the number of associations supporting the expected association divided by the total number of associations for the variable [27]. Two variables have a negative association when the value of one variable increased while another decreased; when two variables both increase a positive association is shown. Based on the percentage of studies supporting the association, the associations were labeled: 0–33% → no association (0); 34–59% → indeterminate or inconsistent (?); 60–100% → positive or negative association (+ or −). If more than 4 studies supported the association in the same direction, it was labeled 00, ??, ++, or −−. The ?? code indicated that a variable has been frequently studied with considerable lack of consistency in findings [27].

## 3. Results

**Figure 1:**
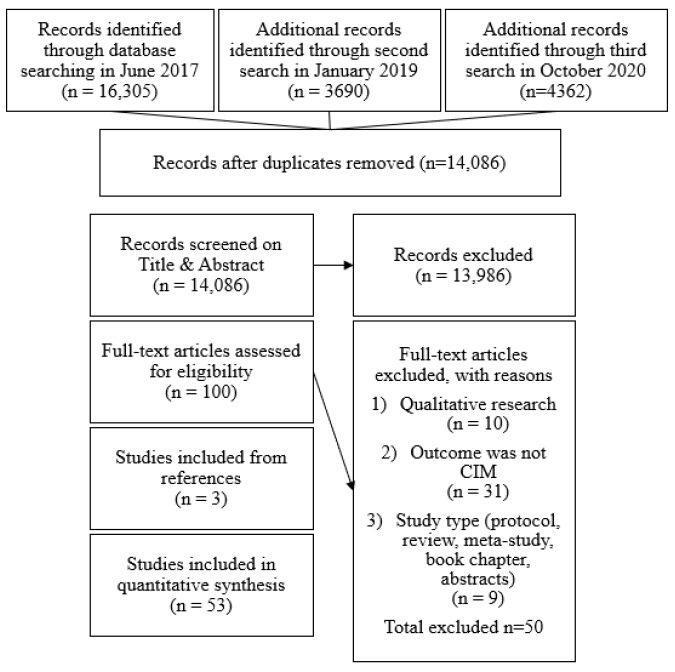
PRISMA flow chart of inclusion and exclusion of studies.

From the 100 full-text articles assessed for eligibility, 53 studies of children’s IM were reviewed. The process of inclusion and exclusion of studies in this review is described in Figure 1 using the preferred reporting items of systematic reviews and meta-analyses PRISMA [26]. The PRISMA checklist [28] and descriptions of included studies can be found in Supplementary Tables S1 and S2.

Studies examined children in the age range of 5 to 17 years old. Most study data originated from Australia (*n* = 15), followed by Canada (*n* = 8), Finland (*n* = 5), and New Zealand (*n* = 4). Three studies originated from Italy, Portugal, Spain, and United States, respectively. These were followed by Belgium, Hong Kong with two studies, respectively, and Austria, Bangladesh, Germany, Norway, Sweden, and United Kingdom each contributed one study. Most studies (48 studies; 91%) were published in 2011 or later. Three studies (6%) were published between 2006–2010 and two studies (4%) were published between 2000–2005. Most studies were cross-sectional in design (50 studies; 94%) and three studies had a longitudinal/prospective design (6% [29,30,31]). Table 1 details characteristics (i.e., year published and country where data originated) of included studies. The criteria for quality assessment and the number (%) of studies scoring points for each criterion can be found in Supplementary Table S3. The overall quality was classified as ‘high’ in 45 studies (85%) and fair in 8 studies (15%). Measures of IM varied amongst the included studies (see Supplementary Table S2). The majority of studies used self-report questionnaires and/or surveys; a little over half of studies (53%) reported both child- and parent-reported data, followed by parent-reported data (38%), and child-reported data (9%). As Bates and Stone reported in their methodological review, various measures of IM exist with no single standardized method [32]. The correlates of children’s IM and their associations are presented through a social-ecological framework including levels for individual, interpersonal, social-environment, and built environment level correlates of children’s IM in Supplemental Table S4.

**Table 1:** Characteristics of studies in systematic review (*n* = 53).

Characteristics	N of Studies (%)	Studies
Country data drawn from		
Australia	15 (28)	Carver et al., 2012 [33], 2013 [34], 2014 [29]; Christian et al., 2014 [35], 2015 [36], 2016 [37]; Curtis et al., 2015 [38]; Foster et al., 2014 [39]; Love et al., 2020 [30]; Schoeppe, Duncan, et al., 2016 [40]; Schoeppe, Tranter, et al., 2016 [41]; Veitch et al., 2008 [42], 2017 [31]; Villanueva et al., 2012 [43], 2014 [44]
Canada	8 (15)	Buliung et al., 2017 [45]; Cervesato et al., 2019 [46]; Delisle Nyström et al., 2019 [47]; Larsen et al., 2015 [48]; Mammen et al., 2012 [49]; Mitra et al., 2014 [50]; Riazi et al., 2019 [51]; Vlaar et al., 2019 [52]
Finland	4 (8)	Broberg, Kyttä, et al., 2013 [53]; Broberg, Salminen, et al., 2013 [54]; Kyttä, 2004 [55]; Kyttä et al., 2015 [13];
New Zealand	4 (8)	Bhosale et al., 2017 [56]; Chaudhury, 2017 [57]; Lin et al., 2017 [58]; Smith et al., 2019 [59]
Italy	3 (6)	Alparone and Pacilli, 2012 [60]; Pacilli et al., 2013 [61]; Prezza et al., 2001 [62]
Portugal	3 (6)	Cordovil et al., 2015 [63]; Lopes et al., 2014 [64]; Santos et al., 2013 [65]
Spain	3 (6)	Ayllón et al., 2019 [66]; Ayllón et al., 2020 [67]; Herrador-Colmenero et al., 2017 [68]
United States	3 (6)	He and Giuliano, 2017 [69]; Janssen et al., 2016 [70]; Wolfe and McDonald, 2016 [71]
Belgium	2 (4)	Ghekiere et al., 2017 [72]; Huertas-Delgado et al., 2018 [73]
Hong Kong	2 (4)	Lam and Loo, 2014 [74]; Loo and Lam, 2015 [75]
Austria	1 (2)	Stark et al., 2018 [76]
Bangladesh	1 (2)	Sharmin et al., 2020 [77]
Germany	1 (2)	Scheiner et al., 2019 [78]
Norway	1 (2)	Fyhri and Hjorthol, 2009 [79]
Sweden	1 (2)	Johansson, 2006 [80]
United Kingdom	1 (2)	Aggio et al., 2017 [81]
Year published		
2016–2020	26 (51)	Aggio et al., 2017; Ayllón et al., 2019; Ayllón et al., 2020; Bhosale et al., 2017; Buliung et al., 2017; Cervesato et al., 2019; Chaudhury, 2017; Christian et al., 2016; Delisle Nystrom et al., 2019; Ghekiere et al., 2017; He and Giuliano, 2017; Herrador-Colmenero et al., 2017; Huertas-Delgado et al., 2018; Janssen et al., 2016; Lin et al., 2017; Love et al., 2020; Riazi et al., 2019; Scheiner et al., 2019; Schoeppe, Tranter, et al. 2016, Schoeppe, Duncan, et al. 2016; Sharmin et al., 2020; Smith et al., 2019; Stark et al., 2018; Veitch et al., 2017; Vlaar et al., 2019; Wolfe and McDonald, 2016
2011–2015	22 (40)	Alparone and Pacilli, 2012; Broberg, Kyttä, et al., 2013; Broberg, Salminen, et al., 2013; Carver et al., 2012, 2013, 2014; Christian et al., 2014, 2015; Cordovil et al., 2015; Curtis et al., 2015; Foster et al., 2014; Kyttä et al., 2015; Lam and Loo, 2014; Larsen et al., 2015; Loo and Lam, 2015; Lopes et al., 2014; Mammen et al., 2012; Mitra et al., 2014; Pacilli et al., 2013; Santos et al., 2013; Villanueva et al., 2012, 2014
2006–2010	3 (5)	Fyhri and Hjorthol, 2009; Johansson, 2006; Veitch et al., 2008
2000–2005	2 (4)	Kyttä, 2004; Prezza et al., 2001

### 3.1. Individual-Level Correlates

Supplementary Table S4 summarizes the associations between correlates of children’s IM that were examined in at least three studies. The review identified six individual-level variables that were examined in three or more studies. Child age and gender were the most examined individual-level correlates. Child age focused around the 10–12 years age range (see Supplementary Table S5). In 81% of the 52 comparisons, older children had greater IM compared to younger children and it was consistently positively associated. Association of child gender was categorized as indeterminate. Varying ethnicity and race variables (e.g., ethnicity, language spoken at home) were consistently positively associated with IM; this association was typically positive for non-minority groups (e.g., White compared to minority ethnicities). Child’s perceived competence, including the child’s safety experience, maturity, and confidence in their IM skills, was consistently positively associated with IM in 60% of the 10 comparisons. Access and ownership of a car was categorized as inconsistent while ownership of a house or child’s access to house keys was consistently positively associated with IM.

### 3.2. Interpersonal-Level Correlates

In this case, 15 correlates were examined in three or more studies. Several variables including parent age, educational level, encouragement (for walking/cycling, or modeling), current physical activity level, travel mode to work, and household structure (e.g., single vs. dual parent household, number of people in family) had no association with IM. Parent gender was categorized as indeterminate along with low socioeconomic status, work status (including employment status and work hours (e.g., parents, mother, father)), parents’ perception of active school travel benefits, and parents’ supportive policies regarding independent play and travel (e.g., parents allow child to walk/cycle to a friend’s house, parents allow child to play anywhere within neighbourhood). Of the 31 comparisons, 77% were consistently positive for having siblings and higher IM. Birth order (referring to a child’s rank by age amongst their siblings) was also positively associated with IM. Additionally, parents’ attitude toward IM and their perception of child’s confidence were consistently positively associated with IM.

### 3.3. Social Environment-Level Correlates

In this case, 12 social environment-level variables appeared in three or more studies. For child-level correlates, children’s positive perceptions of safety (e.g., park is safe, live in a safe area to walk alone) had an inconsistent association with IM while children’s negative perceptions of safety (e.g., cars speeding, crime) and perceptions of social norms (e.g., lots of other children walking or cycling to school, lots of children their own age to hang out with) were categorized as having no association. Only children’s interest (e.g., perception of fun things to do at local park, and children enjoyed walking and cycling) was positively associated. Parents’ positive perceptions of safety in the neighbourhood, parents’ negative perceptions of safety in the neighbourhood, concern about stranger danger, and perceptions of social cohesion were categorized as indeterminate association. Additionally, no consistent association was found for parental perceptions of social norms. Variables found to have no association included parental concern of crime and informal social control. Only parental concern around traffic (e.g., too much traffic around home, heavy traffic around school) was consistently negatively associated with children’s IM (76% of the 21 comparisons).

### 3.4. Built Environment-Level Correlates

In this case, 19 environment-level variables were reported three or more times. Several broad categories of correlates, including destination density, road density, and population density were not associated with IM. Housing/residential density was the only density variable that was consistently positively associated with IM. All destination variables including walking and cycling infrastructure, green space, and ‘other local destinations’ (encompassing destinations such as shopping, recreation, and community centres) were categorized as having no association with IM. Design components such as type of housing (e.g., single-family, multi-family, condominium), and degree of urbanization of an area were consistently categorized as indeterminate. The broad category of urbanization encompassed varying degrees of urbanization—each type of urbanization (e.g., rural, suburban, urban) was associated with IM in various studies, but without a consistent association. The aesthetic quality of the neighbourhood was not associated with IM. Walkability was categorized as indeterminate (e.g., 400 m vs. 1600 m of child’s home). Access to outdoor space, walking, and cycling were positively associated with children’s IM. The only variable that was consistently positively associated with IM was length of residency in one’s home. The diversity variable—land use mix—had no association with children’s IM. While socioeconomic status/neighbourhood socioeconomic deprivation was inconsistently associated. Distance was the second most frequently studied built environment-level variable after degree of urbanization. Shorter distances were consistently positively associated with IM in 90% of the 30 comparisons. Mother’s distance deviation (distance deviation of the child’s school location from mother’s commute) to work was positively associated and in contrast, no association was found with distance/deviation to father’s work. Finally, proximity to green space was positively associated with children’s IM.

## 4. Discussion

Children’s IM has seen a dramatic generational decline which is concerning as IM provides a variety of physical, social, and personal development benefits [12]. Identifying correlates of children’s IM will help inform intervention and policy development. Findings highlighted an acceleration in the number of studies examining correlates of IM over the last decade. This review aimed to comprehensively identify the correlates of IM using a social-ecological framework to map these correlates across individual, interpersonal, social, and built environment levels. As in previous reviews [21,22,23], significantly associated correlates were found at every level of the social-ecological framework highlighting the complexity of children’s IM. In addition, this highlights the need for interventions to target multiple levels to be effective in influencing IM [24].

A wide array of methods was used to measure IM, the most common being parent and child questionnaire or surveys. In line with Bates and Stone’s (2015) methodological review, this review found variability in measurement of IM including use of Hillman’s mobility licenses (i.e., children can travel alone) (1) home from school, (2) to destinations other than school, (3) cross main roads, (4) cycle on main roads, (5) travel on the bus, (6) go out after dark [9], modified mobility licenses (either more or less than six mobility licenses), questions assessing accompaniment to/from school, or to a variety and number of destinations, as well as variability in whom the data were collected from including child-report and/or parent-report [32]. Additionally, IM was described in various ways including, children’s ‘autonomy’ or children being ‘unescorted’ or ‘unaccompanied’. Based on the heterogeneity of measures, methods, and terms used to examine children’s IM, perhaps a standardized measure of IM should be considered in order to facilitate comparisons across global studies as well as considering consistent terminology that could aid in searching for other IM studies.

An interesting result of this review was the lack of consistency seen across the included studies. Of the total 52 variables included, 36 variables demonstrated no association (37%) or indeterminate associations (33%). Associations of these correlates with IM varied across studies (some significant, others not). While some variables were categorized as no association or indeterminate, it may be difficult to rule these out completely especially considering varying measurement methods, how IM was operationalized (e.g., IM licenses vs. whether accompanied to various destinations), and different analyses (e.g., bivariate vs. multivariate) across included studies. These null or inconsistent associations may have been influenced by the sample being examined (e.g., child age, parent vs. child reported data) as well as moderating/confounding effects that were not considered in analyses. Furthermore, it should be considered that several studies used data from larger studies (e.g., RESIDE study [36,37], TREK study [39,44]) and varied in sample size and characteristics which may have influenced the odds of inconsistent findings. Findings that were supported by multiple studies with consistent associations should therefore be acknowledged.

One of the most frequently measured individual-level variables was child age. Unsurprisingly, as children become older, they may gain more maturity, safety skills, and gain competence in their own abilities which may help increase their IM [9]. Additionally, children’s perceived competence was consistently and positively associated with IM. This highlights a need to build children’s competence and confidence in IM and skills associated with it, such as traffic, road, and cycling safety skills. Schools may also influence children’s IM through policies promoting active school travel (e.g., school travel plans), offering cycling and road safety workshops, and promoting further drop-off zones for parents dropping off children [82]. Ethnicity and/or race correlates (specifically, white compared to minority ethnicities) were consistently positively associated with IM, which may point to cultural factors having significant influence on IM. These cultural factors may impact household structures, perceptions of safety and danger, and social norms, which may in turn impact whether families are receptive toward IM. Ethnicity and race have been found to be influential for active school travel [83] and physical activity [27,84,85]. While ethnicity and race variables were associated with IM in this review, the studies varied in ethnicities measured and ethnicity/race was not examined in many studies. Future interventions may consider focusing on child’s skill level, confidence, and competence to be independently mobile and parents’ perceptions of these factors for children’s IM.

Interestingly for interpersonal variables, only parents’ perception of their child’s confidence and their attitude toward independent mobility were consistently positively associated with the freedom to be independently mobile. Unsurprisingly, parents who believed their children were capable of being independent and valued IM and its benefits, had children who had higher IM. Parents’ role as ‘gatekeepers’ to children’s IM should not be ignored. A review by Carver et al. (2008) highlighted parents’ influence on children’s walking and cycling in the neighbourhood and another review by Lee et al. (2015) found parents’ perceived safety concerns was a barrier to children’s independent active free play [86,87]. Moving forward, it may be important to support parents through municipal, provincial, and national policies that encourage or facilitate IM. For example, Utah was the first state in the United States to amend the definition of ‘neglect’ to support children’s independent travel and play [88].

Positive associations with presence of sibling(s) also highlights the importance of children traveling in groups which may help lessen children’s and parents’ concerns [37]. The lack of research on the impacts of friends/peers on children’s IM (e.g., studies asked if child traveled alone or with peer, but not specifically examined) was notable and may be a useful area of future study as traveling with peers can facilitate IM [89].

Only two social environment level correlates were associated with IM: parental concern about traffic and children’s interest (e.g., perception of fun things to do at local park). Interventions should target the built environment particularly in relation to traffic calming measures and traffic safety to help lessen parental concerns as well as providing children with interesting and safe places to travel to and play. At the same time, interventions may need to include a focus on risk reframing. For example, statistically, children are at greater risk of dying as a passenger when travelling in a motor vehicle than being hit by a vehicle when walking or cycling [90,91,92].

Of the many built environment correlates, only six variables showed a consistent positive association: housing/residential density; length of residency in one’s home; access to outdoor walking spaces, walking, cycling; shorter distances; distance/deviation to mother’s work; and proximity to green space. Higher housing/residential density may allude to the availability of children to play with, more ‘eyes on the street’ to provide informal surveillance, while length of time in one’s home may contribute to perceptions of safety, knowledge of the environment, feelings of community, and point to financial stability [93] which may bolster IM. With greater participation of women in the workforce [94], including mothers, increased working hours outside the home, and distance and deviation from commute to child’s school [69], may consequently lead to more opportunities for children to be independently mobile through necessity. Finally, shorter distances, accessibility to green space and outdoor spaces (including for walking and cycling) emphasize the need for urban planners to take these features into consideration when building child-friendly cities. As demonstrated, distance is highly influential for IM and therefore destinations and housing type may be important simply because of proximity to a child’s home (e.g., park across the street from child’s home, recreation centre close by) [95].

This review identified a number of social-ecological correlates and given the diversity of these correlates there is a need for multi-level and multi-sectoral interventions. Some individual-level correlates such as a child’s perception of their competence may be an important focus for future IM promotion initiatives; helping children build the skills and capacity to navigate the environment safely is important. However, the social environment-level and built-environment level may be key areas of focus as they clearly interact. Modification of some individual-level characteristics (e.g., age) may not be possible; instead implementing certain built-environment features that specifically target concerns around traffic and helping children navigate how to safely travel in their respective environments will go a long way to boosting children’s confidence in their abilities, parents’ perceptions of their child skills, and help decrease worries about children being independently mobile. Parents who valued and had a positive attitude towards IM reported their children having higher IM. Addressing child and parental concerns and perceptions of the social environment may be a necessary step in helping reverse the decline in IM. Efforts may need to focus on creating child-friendly environments where the norm is children traveling and playing outdoors, either independently or with their peers. More importantly, this review has highlighted a lack of studies examining correlates of IM at the policy level. Future research and interventions may wish to focus on the influence of policies at the national, provincial, municipal, and/or community level.

### Strengths and Limitations

Strengths of this review included a thorough search of multiple, popular databases, no limitation of publication date, and that the references of included studies were checked for additional studies. Furthermore, the use of the social-ecological framework to guide the review and categorize the correlates of IM was a strength. Taking a social-ecological approach acknowledges the interrelatedness of these identified correlates and points to the importance of considering them in concert to positively impact IM. A limitation of this review is that only correlates examined in three or more studies were included in the final correlates table as per the protocol outlined by Sallis and colleagues [27]. As such, several variables that were examined in less than three studies were not included, but worth mentioning. For example, bicycle ownership was positively associated with girls’ IM [43,44]. Bicycles may provide an alternative, often faster ways, for children to travel to destinations especially those that are further away. Additionally, dog walking and ownership [35,37] and cell phone ownership [33,51] were positively associated with IM and may be important tools that facilitate IM. Dog ownership [96,97], and cell phone ownership [98] may help address child and parental concerns about children traveling alone in the neighbourhood. Location (e.g., Vancouver, BC; Ottawa, ON; Trois-Rivieres, QC) was examined in two studies [47,51] and positively associated with children’s IM which may allude to the influences of urbanization, population density, availability of destinations, and cultural variations that can impact IM. Finally, seasonality (i.e., winter) was negatively associated with IM in one study [79]. Seasonality may be a natural environment variable that should be considered as it may either facilitate (e.g., sunny, warm weather) or impede (e.g., icy conditions, harsh conditions) IM especially if parents or children perceive unsafe conditions. 

Additionally, this review was limited in geographical span, included only English language studies, and focused on children’s independent mobility as the main outcome, which may have resulted in exclusion of some literature and a narrower geographical range. While the studies came from diverse locations around the globe (e.g., North America, Europe, Australia, etc.), most of these countries are considered high income countries [99]. Therefore, the view and understanding of the correlates of children’s IM are highly limited by the context in which they are examined. Future research should examine correlates of IM in low and middle-income countries to better understand context-specific barriers and facilitators of IM. Additionally, future studies are needed to examine correlates of IM more robustly with the inclusion of mediators and moderators and how IM itself may play a role as a mediator/moderator of other health behaviours. This review highlighted that the vast majority of studies examining correlates of IM are cross-sectional in design. Future research to prospectively examine IM as the influence of these correlates may change as a child becomes older (e.g., child’s skills and competence may improve, changes in perceptions of safety) and is needed in the literature. Despite these limitations, this review identifies a range of correlates that may help future researchers tailor interventions.

## 5. Conclusions

Children’s IM may be an important facilitator for PA, while also positively influencing children’s physical, social, and personal development. A variety of correlates were consistently associated with IM and represented all levels of the social-ecological framework and may represent focus areas for future IM policy, practice, and research. Variables that were classified as having an indeterminant or inconsistent association with IM require further study. Given the range of inter-related correlates identified interventions to promote children’s IM likely will need to adopt a multi-level and multi-sectoral approach. However, given the evidence, a focus on building children’s confidence and skills at the individual level, helping parents gain confidence in their child’s abilities to navigate the environment and addressing parental concerns around traffic safety, increasing understanding about benefits of IM, and creating environments where children have shorter distances to travel to reach a variety of destinations of interest (e.g., near green space) should be prioritized. Finally, race and ethnicity and the social and cultural norms tied in with them, are important contextual factors that must be accounted for in future research and practice. Efforts to increase IM should consider how all children (e.g., children with a disability, children of different cultural identities, children living in developing countries) can access safe opportunities for IM, and consider how we, as a society, can equitably support children’s freedom to be independently mobile.

## Data Availability

No new data were created or analyzed in this study. Data sharing is not applicable to this article.

## Supplementary Materials

The following supporting information can be downloaded at: https://www.mdpi.com/article/10.3390/ijerph19031604/s1, Table S1. PRISMA Checklist; Table S2: Descriptions of included studies (*n* = 53); Table S3: Summary of criteria for quality assessment of included studies; Table S4: Associations of social-ecological correlates of children’s independent mobility; Table S5: Sample age ranges of included studies (*n* = 53).
