# Supplementary material for: Social-Ecological Correlates of Children’s Independent Mobility: A Systematic Review
Source: Int J Environ Res Public Health. 2022 Jan 30;19(3):1604. doi: 10.3390/ijerph19031604 (PMC8835222; doi:10.3390/ijerph19031604)
Supplement: Supplementary file 1 [file ijerph-19-01604-s001.zip › ijerph-1484658-supplementary.pdf]

Supplementary Files

Table S1. PRISMA Checklist

Section and Topic	Item #	Checklist item	Location where item is reported
**TITLE**			
Title	1	Identify the report as a systematic review.	Page 1
**ABSTRACT**			
Abstract	2	See the PRISMA 2020 for Abstracts checklist.	1
**INTRODUCTION**			
Rationale	3	Describe the rationale for the review in the context of existing knowledge.	1-2
Objectives	4	Provide an explicit statement of the objective(s) or question(s) the review addresses.	2
**METHODS**			
Eligibility criteria	5	Specify the inclusion and exclusion criteria for the review and how studies were grouped for the syntheses.	3
Information sources	6	Specify all databases, registers, websites, organisations, reference lists and other sources searched or consulted to identify studies. Specify the date when each source was last searched or consulted.	3
Search strategy	7	Present the full search strategies for all databases, registers and websites, including any filters and limits used.	3
Selection process	8	Specify the methods used to decide whether a study met the inclusion criteria of the review, including how many reviewers screened each record and each report retrieved, whether they worked independently, and if applicable, details of automation tools used in the process.	3

Section and Topic	Item #	Checklist item	Location where item is reported
Data collection process	9	Specify the methods used to collect data from reports, including how many reviewers collected data from each report, whether they worked independently, any processes for obtaining or confirming data from study investigators, and if applicable, details of automation tools used in the process.	3
Data items	10a	List and define all outcomes for which data were sought. Specify whether all results that were compatible with each outcome domain in each study were sought (e.g. for all measures, time points, analyses), and if not, the methods used to decide which results to collect.	3-4
	10b	List and define all other variables for which data were sought (e.g. participant and intervention characteristics, funding sources). Describe any assumptions made about any missing or unclear information.	3-4
Study risk of bias assessment	11	Specify the methods used to assess risk of bias in the included studies, including details of the tool(s) used, how many reviewers assessed each study and whether they worked independently, and if applicable, details of automation tools used in the process.	3-4
Effect measures	12	Specify for each outcome the effect measure(s) (e.g. risk ratio, mean difference) used in the synthesis or presentation of results.	4
Synthesis methods	13a	Describe the processes used to decide which studies were eligible for each synthesis (e.g. tabulating the study intervention characteristics and comparing against the planned groups for each synthesis (item #5)).	4
	13b	Describe any methods required to prepare the data for presentation or synthesis, such as handling of missing summary statistics, or data conversions.	4
	13c	Describe any methods used to tabulate or visually display results of individual studies and syntheses.	4
	13d	Describe any methods used to synthesize results and provide a rationale for the choice(s). If meta-analysis was performed, describe the model(s), method(s) to identify the presence and extent of statistical heterogeneity, and software package(s) used.	4

Section and Topic	Item #	Checklist item	Location where item is reported
	13e	Describe any methods used to explore possible causes of heterogeneity among study results (e.g. subgroup analysis, meta-regression).	n/a
	13f	Describe any sensitivity analyses conducted to assess robustness of the synthesized results.	n/a
Reporting bias assessment	14	Describe any methods used to assess risk of bias due to missing results in a synthesis (arising from reporting biases).	n/a
Certainty assessment	15	Describe any methods used to assess certainty (or confidence) in the body of evidence for an outcome.	3; Supplemental file S.3
**RESULTS**			
Study selection	16a	Describe the results of the search and selection process, from the number of records identified in the search to the number of studies included in the review, ideally using a flow diagram.	4
	16b	Cite studies that might appear to meet the inclusion criteria, but which were excluded, and explain why they were excluded.	4
Study characteristics	17	Cite each included study and present its characteristics.	5-6; Supplemental file S.2 and S.4
Risk of bias in studies	18	Present assessments of risk of bias for each included study.	n/a
Results of individual studies	19	For all outcomes, present, for each study: (a) summary statistics for each group (where appropriate) and (b) an effect estimate and its precision (e.g. confidence/credible interval), ideally using structured tables or plots.	Supplemental file S.4
	20a	For each synthesis, briefly summarise the characteristics and risk of bias among contributing studies.	n/a

Section and Topic	Item #	Checklist item	Location where item is reported
Results of syntheses	20b	Present results of all statistical syntheses conducted. If meta-analysis was done, present for each the summary estimate and its precision (e.g. confidence/credible interval) and measures of statistical heterogeneity. If comparing groups, describe the direction of the effect.	Supplemental file S.4
	20c	Present results of all investigations of possible causes of heterogeneity among study results.	n/a
	20d	Present results of all sensitivity analyses conducted to assess the robustness of the synthesized results.	n/a
Reporting biases	21	Present assessments of risk of bias due to missing results (arising from reporting biases) for each synthesis assessed.	n/a
Certainty of evidence	22	Present assessments of certainty (or confidence) in the body of evidence for each outcome assessed.	Supplemental file S.3
**DISCUSSION**			
Discussion	23a	Provide a general interpretation of the results in the context of other evidence.	7-9
	23b	Discuss any limitations of the evidence included in the review.	9-10
	23c	Discuss any limitations of the review processes used.	9-10
	23d	Discuss implications of the results for practice, policy, and future research.	7-10
**OTHER INFORMATION**			
Registration and protocol	24a	Provide registration information for the review, including register name and registration number, or state that the review was not registered.	3
	24b	Indicate where the review protocol can be accessed, or state that a protocol was not prepared.	3
	24c	Describe and explain any amendments to information provided at registration or in the protocol.	n/a

Section and Topic	Item #	Checklist item	Location where item is reported
Support	25	Describe sources of financial or non-financial support for the review, and the role of the funders or sponsors in the review.	12
Competing interests	26	Declare any competing interests of review authors.	12
Availability of data, code and other materials	27	Report which of the following are publicly available and where they can be found: template data collection forms; data extracted from included studies; data used for all analyses; analytic code; any other materials used in the review.	Supplemental files S2-S4

Table S2. Descriptions of included studies (n=53)

Author<br>Year<br>Country<br>*Data from…*	Study Design	Parent Sample	Child sample	Variables / Measurement	Results
Aggio et al.<br>2017<br>United Kingdom<br>*MCS*	Cross-sectional	n=3856*	7 years	**Outcome variable**<br>CIM (play outside without close supervision)<br>Physical activity<br>**Measurement**<br>Survey; Waist-worn accelerometery<br>**Independent variable(s)**<br>Socio-demographics; behavioural (sedentary behaviour); environmental (proximity to friends and family); social (Strengths and Difficulties Questionnaire)<br>**Measurement**<br>Survey	Children who are older, living in poverty, who live in proximity to friends and family, are white British have, and have fewer pro-social behaviours, and more externalizing conduct problems, and fewer internalizing problems, had greater odds of having independent outdoor play.
Alparone et al.<br>2012<br>Italy	Cross-sectional	n=313*<br>Mothers<br>Mean age 37.50 years	8-10 years<br>G (51.4%); B (48.6%)	**Outcome variable**<br>CIM index<br>**Measurement**<br>Questionnaire<br>**Independent variable(s)**<br>Socio-demographics; Maternal perception of social danger; positive potentiality of outdoor autonomy; neighbourhood relations; sense of community;<br>**Measurement**<br>Questionnaire	Mother's perception of positive potentiality of outdoor autonomy was + associated with CIM. Social danger perception and birth order were – associated and child age was + associated with CIM.
Ayllón<br>2019<br>Spain<br>*CAPAS-Ciudad*	Cross-sectional	n=1450*<br>Mother mean age 43.02±5.03<br>Father mean age 45.38±5.43	n=1450<br>9-11 years old;<br>Grades 4, 5, 6<br>Mean age 10.53±0.90<br>G (53.2%); B (46.8%)	**Outcome variable**<br>CIM (CIM to/from school)<br>**Measurement**<br>Questionnaire<br>**Independent variable(s)**<br>Parent reported: Socio-demographics; child autonomy, perceived difficulty for	Parents' willingness for CIM to school, parents' perception of child autonomy, child having house keys, year in school, position in the family were + associated with CIM to school. While parents' perceived difficulty for CIM to school was – associated with CIM.

				CIM, parents' willingness for CIM, frequency of CIM for outdoor leisure activities, having house keys, perceived dangers<br>**Measurement**<br>Questionnaire	Children in year 6 had greater CIM compared to children in lower grades.
Ayllón<br>2020<br>Spain<br>*CAPAS-Ciudad*	Cross-sectional		n=1106*<br>8-12 years old;<br>Mean age 10.6±2.04<br>G (48.6%); B (51.4%)	**Outcome variable**<br>CIM (CIM to/from school)<br>**Measurement**<br>Questionnaire<br>**Independent variable(s)**<br>Child reported: Socio-demographics; perceived difficulty for CIM, perceived distance to school, attitude for CIM to school, willingness for CIM; house keys, CIM for outdoor leisure<br>**Measurement**<br>Questionnaire	The study highlighted that perceived difficulty of CIM to school (-), attitudes and willingness for CIM to school (+) were associated with CIM to and/or from school. A shorter perceived distance home to school was associated with greater CIM.
Bhosale<br>2017<br>New Zealand	Cross-sectional	n=500*<br>Mean age 43.9±5.8 years<br>F=373; M=118	n=544*<br>12.2 ±0.6 years<br>G=272; B=257	**Outcome variable**<br>CIM (mobility licenses and index)<br>**Measurement**<br>Questionnaire<br>**Independent variable(s)**<br>Socio-demographics; active transport; organized activities; bicycle/car ownership; parents' past IM behaviour<br>**Measurement**<br>Questionnaire	Parents had greater IM than their children and a greater number of mobility licences. Bike ownership increased and number of organized activities decreased generationally.
Broberg et al. (a)<br>2013<br>Finland	Cross-sectional		n=1837*<br>5th and 7th grade<br>10-12 years; 13-15 years<br>B (>50%)	**Outcome variable**<br>CIM (accompaniment to affordances)<br>**Measurement**<br>Survey<br>**Independent variable(s)**<br>Environmental likeability, Bullerby grid; GIS-based environmental measures<br>**Measurement**<br>Survey<br>Mapping activity	Increasing housing density and population around an affordance was + associated with accessing the affordance independently. Floor area ratio was – associated with independent access to affordances.

Broberg et al. (b)<br>2013<br>Finland	Cross-sectional		n=901*<br>Year 5 (47%)<br>11 years;<br>Year 8 (53%)<br>14 years	**Outcome variable**<br>CIM (accompaniment to each meaningful place)<br>**Measurement**<br>softGIS survey<br>**Independent variable(s)**<br>Socio-demographics, physical environment measured factors (GIS)<br>**Measurement**<br>softGIS survey	Single family housing, longer distances from public transport and sports facilities, and dense residential structures were + associated with CIM. Big buildings and public transport hubs and increasing distance from home to school were – associated with CIM.
Buliung<br>2017<br>Canada<br>*BEAT*	Cross-sectional	n=651* (to school)<br>n=708* (from school)	n=651* (to school)<br>n=708* (from school)<br>9-13 years<br>Mean age 11 years<br>G (50%); B (50%)	**Outcome variable**<br>CIM (travel independently/escorted)<br>**Measurement**<br>Activity travel survey<br>**Independent variable(s)**<br>Socio-demographics; environment; parent and child perceptions of safety<br>**Measurement**<br>Activity travel survey; route mapping	A number of variables are associated with CIM including distance, age, gender, traffic volume and flexible parental work schedules.
Carver et al.<br>2012<br>Australia	Cross-sectional	n=891*	n=688*<br>Primary schoolchildren<br>n=430<br>G (52%); M(48%)<br>Mean age 10.4±1.2<br>Secondary schoolchildren<br>n=258<br>G (48%); B (52%)<br>Mean age 13.7±1.0<br>School year 3-10	**Outcome variable**<br>CIM (travel mode to/from school/weekends, accompaniment, mobility licences score)(0-6 scale)<br>**Measurement**<br>Policy Studies Institute (PSI) international CIM Study Child & parent surveys<br>**Independent variable(s)**<br>Socio-demographics, Settlement types (urban/rural), mobility licences<br>**Measurement**<br>PSI Child & parent survey	CIM had no significant differences in settlement type (urban/rural). Mobile phone ownership + associated with mobility licences in boys attending urban primary schools. Mobility licences were + associated odds of walking/cycling independently in boys and primary school girls (not significant for girls in rural areas). Access to outdoor play spaces was + associated with mobility licences for urban boys and rural primary girls.
Carver et al.<br>2013<br>Australia	Cross-sectional	n=1239*<br>Mothers (88%)	n=784* Primary schoolchildren<br>Mean age=10.4±1.2<br>n=455* Secondary schoolchildren<br>Mean age=13.7±1.0	**Outcome variable**<br>CIM (mobility licences(0-6 scale); actual mobility<br>**Measurement**<br>Child & parent questionnaire<br>**Independent variable(s)**	Mobility licences were + associated with higher levels of CIM on non-school journeys (both primary/secondary) and walking/cycling independently to school (primary schoolchildren).

			10-12 years<br>English and Australian children<br>G (50%); B (50%)	Urbanization (rural, urban); household car access; mobility licenses<br>**Measurement**<br>Child & parent questionnaire	
Carver et al.<br>2014<br>Australia<br>*SPEEDY*	Longitudinal	n=1121*	n=1121* (T1)<br>G (57%); B (43%)<br>n=491* (T2: 1 year follow-up)<br>G (61%); B (39%)<br>9-10 years	**Outcome variable**<br>CIM (travel mode to school; accompaniment)<br>**Measurement**<br>Child & parent questionnaire<br>**Independent variable(s)**<br>Socio-demographics; environment characteristics (GIS); perceptions of social and physical environment; neighbourhood walkability score; parental rules about child's physical activity<br>**Measurement**<br>Child & parent questionnaire	Boys had higher CIM compared to girls, but difference diminished with age. Longitudinally, land use mix was + associated and proportion of main roads and parental encouragement were – associated with girls' CIM. Number of siblings was + associated with CIM at T1.Parents allowing children to play outside anywhere in neighbourhood was + associated and household car access was – associated longitudinally with boys' CIM.
Cervesato et al.<br>2019<br>Canada	Cross-sectional	n=979*<br>weekday trips;<br>n=315*<br>weekend trips	n=979 weekday trips; n=315 weekend trips<br>9-11 years	**Outcome variable**<br>CIM (independent weekday/weekend trips)<br>**Measurement**<br>Survey: 2011 Quebec Metropolitan Community Origin-Destination data<br>**Independent variable(s)**<br>Socio-demographics, household variables, built environment variables<br>**Measurement**<br>Survey: 2011 Quebec Metropolitan Community Origin-Destination data	Independent trips of children 9-11 years old drops significantly on weekends versus weekday. A number of individual-level variables (age (+), gender (girls +), household variables (number of cars (-), number of persons (-)), and built environment variables (new suburbs, periphery, outlying rural (-); distance (-)) were significantly associated with children's independent trips.
Chaudhury et al.<br>2017<br>New Zealand<br>*KITC*	Cross-sectional	n=240*	n=240*<br>9-12 years	**Outcome variable**<br>CIM (time, origin, destination, travel mode, accompaniment); parental licence of freedom score (1-10 scale)<br>**Measurement**<br>Child travel diaries; Parent telephone interview<br>**Independent variable(s)**	Child age and degree of mobility licences + associated with CIM to a POS. Ethnicity was also significantly associated with independent trip to POS.

				Demographics; Public Open Space (POS) quality/quantity<br>**Measurement**<br>Parent telephone interview; POSAI (environmental audit tool)	
Christian et al. 2014<br>Australia<br>*TREK*	Cross-sectional	n=727*	n=727*<br>10-12 years<br>Years, 5,6,7	**Outcome variable**<br>CIM (15 local destinations, active independent travel)(0-15 scale)<br>**Measurement**<br>Child & parent questionnaire<br>**Independent variable(s)**<br>Socio-demographics; Physical activity, local walking and outdoor play; dog walking<br>**Measurement**<br>Child & parent questionnaire<br>Accusplit AH120 Pedometer	Dog walkers were more independently mobile than non-dog walkers.
Christian et al. 2015<br>Australia<br>*RESIDE*	Cross-sectional	n=305*	n=181<br>8-15 years	**Outcome variable**<br>CIM (parent report; travel to/from various destinations)<br>**Measurement**<br>Parent survey<br>**Independent variable(s)**<br>Physical environment (GIS), social environment (i.e. neighbourhood perceptions)<br>**Measurement**<br>Parent survey	Distance (e.g., to school, local park), perception of an unsafe neighbourhood, and unsupportive parenting social norms were – associated with CIM.
Christian et al. 2016<br>Australia<br>*RESIDE*	Cross-sectional	n=305*	n=181<br>8-15 years<br>mean age<br>10.7(±2.1)	**Outcome variable**<br>CIM (parent report; travel to/from various destinations)<br>**Measurement**<br>Parent survey<br>**Independent variable(s)**<br>Siblings/older siblings; dog ownership<br>Measurement	Having an older sibling, older sibling of the same gender, owning a family dog were + associated with CIM to a variety of destinations.
Cordovil et al. 2015	Cross-sectional	n=1099*<br>Mothers (82%)	n=1099*<br>8-15 years	**Outcome variable**	Age was + associated with CIM. Gender did not influence CIM except in number of

Portugal			Primary n=660<br>G (51%); B (49%)<br>mean age =9.8±1.5<br>Secondary n=439<br>G (57%); B (43%)<br>mean age=13.8±1.6	CIM (mobility licences; travel mode; CIM on weekends)<br>**Measurement**<br>Portuguese version of Policy Studies Institute (PSI) international CIM Child & parent questionnaire<br>**Independent variable(s)**<br>Socio-demographics; distance home to school; school setting<br>**Measurement**<br>PSI Child & parent questionnaire	independent activities done on the weekend. Rural settings were + associated with CIM compared to urban settings. Distance was – associated with CIM, and having no car access was positively associated to CIM.
Curtis et al.<br>2015<br>Australia	Cross-sectional	n=273*	n=273*<br>9-13 years<br>G (64.8%); B (35.2%)	**Outcome variable**<br>CIM (travel mode to school; other locations)<br>**Measurement**<br>Child & parent questionnaire<br>**Independent variable(s)**<br>Socio-demographics; built environment<br>**Measurement**<br>Child & parent questionnaire	Distance to school is associated with CIM. Children who actively travel live closer to school and associated with more metropolitan, denser, and more walkable environments. 800 m ped-shed ratio, densities, and distance to school are associated with CIM.
Delisle Nyström et al.<br>2019<br>Canada<br>*ATIM*	Cross-sectional	n=1699*	n=1699*<br>8-13 years old<br>mean age 10.2±1.0<br>G (n=935); B (n=764)	**Outcome variable**<br>CIM (Hillman mobility licenses; 0-6 scale)<br>**Measurement**<br>Survey<br>**Independent variable(s)**<br>Socio-demographics; area-level SES; urbanization; site<br>**Measurement**<br>Survey	Area-level SES and urbanization were not associated with CIM. For girls, site (Trois-Rivières) was + associated with CIM. Age was + associated with children's independent mobility licenses. Home ownership was + associated with boy's independent mobility licenses. Parental education was not associated with CIM licenses.
Foster et al.<br>2014<br>Australia<br>*TREK*	Cross-sectional	n=1231*	n=1231*<br>10-12 years	**Outcome variable**<br>CIM (15 local destinations; accompaniment) (0-15 scale)<br>**Measurement**<br>Child & parent questionnaire<br>**Independent variable(s)**<br>Socio-demographics; informal social control; parental fear of strangers, social	Parental fear of strangers was – associated with CIM. Informal social control was + associated with CIM.

				and built environment (GIS) characteristics<br>**Measurement**<br>Child & parent questionnaire	
Fyhri et al.<br>2009<br>Norway	Cross-sectional	n=1282*<br>F (55%); M (45%)	Children in age range of 6-12 years	**Outcome variable**<br>CIM (school trip; sport activities; travel mode; accompaniment) (1-9 scale)<br>**Measurement**<br>NTS 2005 travel survey parent report<br>Child survey<br>**Independent variable(s)**<br>Socio-demographics; distance to various destinations; traffic; parents' experience of safety; child's experience of safety<br>**Measurement**<br>NTS 2005 travel survey parent report<br>Child survey	Parents car use, number of cars, and work hours, and distance to school and sports were – associated with CIM. Parents' and children's safety experience on school and leisure trip + associated with CIM. Seasonality was also associated with CIM.
Ghekiere et al.<br>2017<br>Belgium	Cross-sectional	n=1286*<br>Mothers (77%)	Children in age range of 10-12 years	**Outcome variable**<br>CIM (distance child could cycle alone for transport)<br>**Measurement**<br>Parent online questionnaire<br>**Independent variable(s)**<br>Demographics; psychosocial factors; neighbourhood environmental perceptions<br>**Measurement**<br>Parent online questionnaire	Boys had more CIM compared to girls. Parent perceptions of child's cycling and traffic skills, perceptions of neighbourhood traffic safety were + associated with CIM (the latter only true for girls). Age was + associated with CIM. Perceptions of cycling skills + associated with CIM for boys in high urbanized areas. Parental cycling for transport was – associated with CIM among low SES girls.
He et al.<br>2017<br>United States	Cross-sectional	Total school trips n=3172*<br>Total households n=2039<br>Two –parent households n=1491<br>father-headed n=115	School age children	**Outcome variable**<br>CIM (independent trips, alone or with siblings, by non-motorized mode; child driving him or herself; child travelling by bus)<br>**Measurement**<br>2001 Southern California Association of Governments (SCAG) Post Census Regional Household Travel Survey<br>**Independent variable(s)**	Distance to parents' work was + associated with CIM to school, especially for distance to mothers' work place and children walking to school independently. Child age is + associated with walking/cycling independently and driving. Number of siblings and car availability was – associated with CIM and active commuting. Children from low SES households are more likely to take the bus then be chauffeured by the

		mother-headed n=418<br>Other types n=15		Socio-demographics; Parent employment/working arrangements; distance home, school, parents' workplace<br>**Measurement**<br>2001 SCAG Post Census Regional Household Travel Survey	mother. Single family housing – associated with taking the bus and multi-family residential land + associated with walking/cycling alone or with siblings.
Herrador-Colmenero<br>2017<br>Spain	Cross-sectional		n=652*<br>F (n=313); M (n=339)<br>6-12 years	**Outcome variable**<br>CIM (accompaniment on journey to school)<br>**Measurement**<br>Questionnaire<br>**Independent variable(s)**<br>Socio-demographics; accompaniment mode, safety perceptions, mode of commuting<br>**Measurement**<br>Questionnaire	Children who were older were more likely to travel independently to school and had better safety perceptions compared to younger children. Accompanied children and less understanding of safety issues compared to children who actively commuted independently.
Huertas-Delgado<br>2018<br>Belgium<br>*BEPAS*	Cross-sectional	n=291*<br>41.4±8.9 years<br>Mothers (76.5%)	n=291*<br>12-15 years<br>Mean age 13.2±1.0 years<br>G (54.7%)	**Outcome variable**<br>CIM (time spent independently traveling for walking, cycling public transport)<br>**Measurement**<br>Questionnaire<br>**Independent variable(s)**<br>Socio-demographics; perceived neighbourhood environmental factors<br>**Measurement**<br>Questionnaire	Parents reported greater negative perception of traffic and crime related safety. For adolescents, parents' traffic and crime related perceptions were associated with adolescents' IM, but not active IM.
Janssen et al.<br>2016<br>United States	Cross-sectional	n=497*<br>American parents	Children in age range of 6.9-11.9 years	**Outcome variable**<br>CIM (range child can travel; accompaniment)<br>**Measurement**<br>Parent survey<br>**Independent variable(s)**<br>Socio-demographics; interpersonal/family level characteristics; perceptions of neighbourhood safety<br>**Measurement**	Child age and parents' perception of neighbourhood safety were + associated with CIM. Parents' fear of crime was – associated with CIM.

				Parent survey	
Johansson et al.<br>2006<br>Sweden	Cross-sectional	n=357*<br>Mothers (78%)<br>Travel diary completed<br>n=248	Children in age range of 8-11 years<br>Mean age = 9.6 years<br>G (50%); B (50%)	**Outcome variable**<br>CIM (Travel mode)<br>**Measurement**<br>Parent questionnaire; travel diary<br>**Independent variable(s)**<br>Socio-demographics; environmental trust; interpersonal trust; social environment (sense of community); physical environment (traffic, foot/cycle paths)<br>**Measurement**<br>Parent questionnaire	Parent attitude toward independent travel (+), traffic environment (+), car access (-), and child age (+) were associated with children's independent journeys.
Kytta et al.<br>2004<br>Finland	Cross-sectional	n=223*<br>Finland n=80<br>Belarus n=147	n=223*<br>8-9 years<br>Finland n=80<br>Mean age=8.5 years<br>Belarus n=147<br>Mean age=8 years	**Outcome variable**<br>CIM (mobility licences; actual mobility)<br>**Measurement**<br>Child questionnaire & interviews; parent questionnaire<br>**Independent variable(s)**<br>Socio-demographics; environment characteristics (community type)<br>**Measurement**<br>Child questionnaire & interviews; parent questionnaire	The degree of mobility licence was + associated with children's actual mobility (Finnish data). Degree of urbanisation was – associated with mobility licences. Gender (boy) was + associated with actual mobility (Belarusian data).
Kytta et al.<br>2015<br>Finland	Cross-sectional	Second sample n=306*	n=821*<br>7-15 years<br>Second sample n=306*<br>8-10 years	**Outcome variable**<br>CIM (mobility licences, actual mobility; independent weekend activities)<br>**Measurement**<br>Child & parent survey<br>**Independent variable(s)**<br>Socio-demographics; settlement type;<br>**Measurement**<br>Child & parent survey	Children in suburban and large towns had higher CIM than inner city, small town, and rural children. Child age + associated with CIM. No significant association between genders, except girls in small town setting had fewer mobility licences. Access to a car was – associated with CIM.
Lam et al.<br>2014<br>Hong Kong	Cross-sectional	n=1672*<br>Families<br>Parent-report	n=2110<br>6-12 years	**Outcome variable**<br>CIM (accompaniment to school; dichotomous variable yes/no)<br>**Measurement**<br>Travel Characteristic Survey 2002	Child age was positively associated with CIM while distance was negatively associated with CIM. Median household income (low SES +), family structure (single parent +), mother's working status (+), employment of

				**Independent variable(s)**<br>Socio-demographics, family, environmental characteristics<br>**Measurement**<br>Travel Characteristic Survey 2002	a domestic helper (-), neighborhood settlement type (rural +), and density of school places.
Larsen et al.<br>2015<br>Canada	Cross-sectional	n=1016<br>Final sample with complete data n=559*	Children of elementary school age	**Outcome variable**<br>CIM (walking independently)<br>**Measurement**<br>Telephone survey on school travel<br>**Independent variable(s)**<br>Socio-demographics; social environment; environment characteristics (GIS)<br>**Measurement**<br>Telephone survey on school travel	Child's age was + associated with CIM. Population density was – associated with independent walking. Neighbourhood age was associated with CIM in that location of one's home modifies the relationship between age and CIM. Intersection density was – related to travel mode (walking). Distance was – associated with children walking (vs. driving).
Lin et al.<br>2017<br>New Zealand<br>*KITC*	Cross-sectional	n=239*	n=254*<br>8-13 years	**Outcome variable**<br>CIM (travel mode and accompaniment)<br>**Measurement**<br>Travel diary<br>Parent computer-aided telephone interview<br>**Independent variable(s)**<br>Perceptions of neighbourhood safety, cohesion, connection<br>Built environment<br>**Measurement**<br>Parent computer-aided telephone interview<br>Objective measures ArcInfo 9.3	Parental perceptions of neighbourhood cohesiveness and connectedness were positively associated with CIM. Proximity to school was also associated with independent trips.
Loo et al.<br>2015<br>Hong Kong	Cross-sectional	n=2110	n=2110*<br>6-12 years	**Outcome variable**<br>CIM (independent trips to school)<br>**Measurement**<br>Travel Characteristics Survey 2002<br>**Independent variable(s)**<br>Socio-demographics and environmental characteristics<br>**Measurement**<br>Travel Characteristics Survey 2002	Children from higher SES households were more likely to have less independent trips to school.

Lopes et al.<br>2014<br>Portugal	Cross-sectional	n=1099*	n=1099* (16 schools)<br>9-14 years<br>Grade 3-10	**Outcome variable**<br>CIM<br>**Measurement**<br>International child independent mobility questionnaires<br>**Independent variable(s)**<br>Socio-demographics<br>Psychosocial factors (Fear perceptions, sense of community)<br>Urbanization<br>**Measurement**<br>International child independent mobility questionnaires	Increasing urbanization decreased the odds of independent mobility. Parental fear of traffic and stranger danger were commonly cited concerns for parents.
Love et al.<br>2020	Longitudinal	n=1136*	n=1136*<br>9-12 years<br>G (n=570); B (n=552)	**Outcome variable**<br>CIM (to/from school; binary independent vs. not independent)<br>**Measurement**<br>Survey<br>**Independent variable(s)**<br>Individual level, social capital at neighbourhood/school community level; objective/perceived neighbourhood built environment<br>**Measurement**<br>Survey	A number of factors were significantly associated with change in CIM including mode of travel at baseline (+), number of independent weekend trips (+), and social capital factors like child knowing neighbours well (+) and parents perceiving that neighbours are willing to help each other (+) were associated with increased CIM on the school journey.
Mammen et al.<br>2012<br>Canada	Cross-sectional	n=1016<br>n=490* (lived within 2km of school used for analysis)<br>Parents	6-14 years<br>Unescorted walkers<br>G (52.3%); B (47.7%)	**Outcome variable**<br>CIM (travel mode reported as escorted vs. unescorted)<br>**Measurement**<br>Self-reported survey by Metrolinkx<br>**Independent variable(s)**<br>Socio-demographics<br>Parental attitudes/perceptions toward active school travel<br>**Measurement**<br>Self-reported survey by Metrolinkx	Unescorted children were significantly older, English predominantly spoken at home, and lived closer to school. Parental fears of stranger danger and bullying were associated with children being escorted to school.
Mitra et al.<br>2014	Cross-sectional	n=795*	Grade 5 and 6	**Outcome variable**<br>CIM	Positive parental neighbourhood perceptions of safety increased chances of

Canada<br>*BEAT*		n=686 (valid accelerometry data)	G (53.46%); B (46.54%)	**Measurement**<br>Questionnaire<br>**Independent variable(s)**<br>Socio-demographics, Parental attitudes toward transportation mode; perceptions of neighbourhood environment<br>Physical activity<br>**Measurement**<br>Questionnaire<br>Accelerometry	children's IM. Boys and older children more likely to have more IM than girls and younger children.
Pacilli et al.<br>2013<br>Italy	Cross-sectional	n=589*<br>Parents	n=589*<br>10-15 years<br>G (49%); B (51%)	**Outcome variable**<br>CIM (7 questions; did not refer to school route)<br>**Measurement**<br>Questionnaire (Child)<br>**Independent variable(s)**<br>Socio-demographics, environmental, psychosocial characteristics<br>Sense of community (Child)<br>Parenting style (Parent)<br>**Measurement**<br>Questionnaire (Child & Parent)<br>Parental Styles Scale (Parent)	Age was + associated with IM, while gender (i.e. female) was – associated with IM.
Prezza et al.<br>2001<br>Italy	Cross-sectional	n=251*<br>Mothers<br>Mean age 39	n=251<br>7-12 years<br>Mean age 9.41<br>G (47%); B (53%)	**Outcome variable**<br>CIM (to/from school; outside play; to/from destinations; overall score from 5 partial scores)<br>**Measurement**<br>Semi-structured interview<br>**Independent variable(s)**<br>Neighbourhood relations/sense of community<br>Environmental factors<br>**Measurement**<br>Child's frequentation of peers<br>Neighbourhood Relations Scale<br>Italian Scale of Sense of Community	Older children and boys have significantly higher IM compared to younger children and girls. Greater IM was also experience by children whose mothers had stronger neighbourhood relations, lived in a building with a courtyard, near a park, or in a new neighbourhood.

Riazi et al.<br>2019<br>Canada<br>*ATIM*	Cross-sectional	n=1699*<br>Mothers (80.9%)<br>30-44 years (68.9%)	N=1699*<br>Mean age 10.21±0.98<br>G (55.1%); B (44.9%)	**Outcome variable**<br>CIM (Hillman 6 mobility licenses; CIM index 0-6)<br>**Measurement**<br>Survey (parent)<br>**Independent variable(s)**<br>Socio-demographics, social environment factors, physical and built environment factors<br>**Measurement**<br>Survey (Child and Parent)	Individual correlates of CIM included child grade level (+), language spoken at home (-), car ownership (-), and phone ownership (+). For boys, parental gender was negatively associated with CIM. Parental perceptions of safety and environment were significantly associated with CIM. Location was significantly associated with CIM and suburban environments were (-) associated with boys' independent mobility and walkability was (+) associated with girls' independent mobility.
Santos et al.<br>2013<br>Portugal<br>*SALTA*	Cross-sectional	n=354*<br>Mothers (74%); Fathers (23.2%)	n=354*<br>Mean age 11.63(.85)<br>G (66%); B (44%)	**Outcome variable**<br>CIM (child report visitation/accompaniment of 11 destinations (5 point Likert scale))<br>**Measurement**<br>Survey (Child)<br>**Independent variable(s)**<br>Parental PA<br>Perceptions of neighbourhood safety<br>**Measurement**<br>Survey (Parent)<br>IPAQ	Parents' PA and perception of sidewalk and street safety were significantly associated with children's IM.
Scheiner et al.<br>2019<br>Germany	Cross-sectional	n=605* morning trip; n=594 afternoon trip (described as net sample)<br>*demographics unreported	n=605 morning trip; n=594 afternoon trip<br>(described as net sample)<br>6-11 years<br>Mean age 7.87±1.28	**Outcome variable**<br>CIM (escort mode to and from school)<br>**Measurement**<br>Questionnaire<br>**Independent variable(s)**<br>Sociodemographic, transport, built environment, traffic safety, parental concerns, perceptions, attitudes<br>**Measurement**<br>Questionnaire	A number of individual, interpersonal, social, and built environment level factors were associated with the odds of being escorted to and from school. More built environment variables were associated with the trip to school but not the afternoon trip, while more attitudinal variables were associated with the trip from school.

Schoeppe et al. (a)<br>2016<br>Australia	Cross-sectional	Data drawn from*<br>1991: n=1360<br>1993: n=298<br>2010: n=341<br>2011: n=113<br>2012: n=301	8-13 years*<br>Mean age 10 years<br>1991: n=1273<br>1993: n=476<br>2010: n=421<br>2011: n=131<br>2012: n=305	**Outcome variable**<br>CIM (child actual mobility; parent licences for IM)<br>**Measurement**<br>Surveys (Child & Parent)	Younger children and girls were less likely to travel to from home to school independently. Overall, the findings suggest that IM has declined in Australian children from 1991 to 2012.
Schoeppe et al. (b)<br>2016<br>Australia	Cross-sectional	n=1293* (1164 with complete data)<br>Australian adults<br>Mean age 55.8(15.6)<br>F (48%); M (52%)	8-12 years	**Outcome variable**<br>CIM (adult attitudes on distances child should be able to travel/play outdoors independently)<br>**Measurement**<br>Queensland Social Survey (QSS)<br>**Independent variable(s)**<br>Demographics, adult attitudes<br>**Measurement**<br>Queensland Social Survey (QSS)	More than half of adults (62%) would restrict children's independent travel to <500m from home and 74% of adults would restrict children's independent outdoor play to <500m from home. Women and adults with lower education were more likely to restrict IM (travel & play) to shorter distances (<500m).
Sharmin et al.<br>2020<br>Bangladesh	Cross-sectional	n=151*<br>Majority of parents in 30-45 age range<br>F (62.3%); M (34.4%); other (3.3%)	n=151*<br>10-14 years<br>Mean age 12.5 years<br>G (53%); B (47%)	**Outcome variable**<br>CIM (accompaniment of each trip)<br>**Measurement**<br>Travel diary<br>**Independent variable(s)**<br>Socio-demographics, built environment (geographic and topological data)<br>**Measurement**<br>Questionnaire, travel diary, geographic and topological built environment data	A number of geographic and topological built environment factors are associated with CIM but affect discretionary (angular integration (-), recreational land use (+), and traffic composition along route (+)) and non-discretionary (i.e. step-depth (-), angular connectivity (-); presence of institutional land use (-)) independent trips differently
Smith et al.<br>2019<br>New Zealand<br>*Neighbourhood for Active Kids study*	Cross-sectional	n=931*	n=931<br>5-11 years (52.2%);<br>10-13 years (47.8%)<br>F (49.6%); M (50.4%)	**Outcome variable**<br>CIM (6 licenses)<br>**Measurement**<br>CATI Computer-aided telephone interview mapping survey<br>**Independent variable(s)**<br>Socio-demographics; neighbourhood-built environment measures	No significant differences were observed between objective neighbourhood-built environment measures and parents' reported needs. However, objectively assessed (-) and parent-reported need (+) for cycling infrastructure were associated with parental license.

				**Measurement**<br>CATI Computer-aided telephone interview mapping survey; objective built environment features ArcMap 10.5, Auckland Transport's Open GIS Data	
Stark<br>2018<br>Austria	Cross-sectional	n=380*	n=380*<br>6-9 years<br>G (49%)	**Outcome variable**<br>CIM (mobility licenses)<br>**Measurement**<br>Survey; travel diary<br>**Independent variable(s)**<br>Socio-demographics; parents' occupation; car ownership; frequency of accompaniment to school and travel modes<br>**Measurement**<br>Survey	Parents' attitudes towards active independent mobility influence children's travel patterns as well as distance, age, type of school are also associated.
Veitch et al.<br>2008<br>Australia	Cross-sectional		n=212*<br>8-12 years<br>8-9 years (49%); 10-12 years (51%)<br>G (51%); B (49%)	**Outcome variable**<br>CIM (child report were walk/ride without adult)<br>**Measurement**<br>Survey (Child)<br>Behavioural maps of neighbourhood (Child)<br>**Independent variable(s)**<br>Individual characteristics<br>Distance (direct/computer generated measurement)<br>**Measurement**<br>Survey (Child)<br>Mapping activity (Child)	Older children (vs. younger children) were more likely to go to 3+ places independently. Children in lower SES environments reported higher IM in number of locations visited and distance traveled.
Veitch et al.<br>2017<br>Australia<br>*READI*	Longitudinal<br>Baseline (2007-08)<br>T1 (2010)<br>T2 (2012)	n=311 (T1)*<br>Mothers<br>Mean age 41.3(8.4)	n=311 (T1)*<br>n=207 (T2)*;<br>184/179 analyzed (local destinations/school)<br>5-12 years	**Outcome variable**<br>CIM (child report travel mode for trips to school, active/nonactive, accompaniment; 4 destinations (0-10 scale); walking/cycling to local destinations (0-20 scale))<br>**Measurement**	Parental perceptions of safety, distance to walking and tracks and enjoyment of walking/cycling were longitudinally associated with IM on the school journey. Mother and child's agreement that other children they know walk/cycle to school was also + associated with IM travel.

			Mean age<br>12(2.1)(T1)<br>G (55%); B (45%)	Questionnaire (Child)(T1 & T2)<br>**Independent variable(s)**<br>Objective measures of neighbourhood (GIS)<br>Individual, social, Perceived neighbourhood attributes<br>**Measurement**<br>Survey (Mother)(T1)	
Villanueva et al.<br>2012<br>Australia<br>*TREK*	Cross-sectional	n=1314*	n=1480*<br>10-12 years<br>G (51%); B (49%)	**Outcome variable**<br>CIM (15 activities; child report participation/visitation; parent report accompaniment)(0-15 scale)<br>**Measurement**<br>Questionnaire (2007)(Child & Parent)<br>**Independent variable(s)**<br>Objective environmental factors (GIS)<br>Perceived social, individual, environmental factors (Child & Parent)<br>**Measurement**<br>Questionnaire (2007)<br>Mapping activity (Child)	Parents' perceptions of living near a busy road, decreased odds of children's IM. Girls who lived in well-connected and low traffic neighbourhoods had increased IM. Boys who had access to local recreation or retail destination also experienced higher IM. Boys who lived near shopping centers or community services had lower levels of IM.
Villanueva et al.<br>2014<br>Australia<br>*TREK*	Cross-sectional	n=1314 *	n=1480 *<br>10-12 years<br>G (52%); B (48%)	**Outcome variable**<br>CIM (15 activities; child report participation/visitation; parent report accompaniment)(0-15 scale)<br>**Measurement**<br>Questionnaire (2007)(Child & Parent)<br>**Independent variable(s)**<br>School-specific walkability index (GIS)<br>Perceived social, individual, environmental factors (Child & Parent)<br>**Measurement**<br>Questionnaire (2007)	High walkability was + associated with girls' IM. Parents' and child's confidence in child's ability to walk independently was * associated with higher levels of IM. Parent perceptions of safe neighbourhood road crossings were + associated with boys' IM.
Vlaar et al.<br>2019<br>Canada	Cross-sectional	n=105*<br>Mean age<br>45±5	n=105*<br>Child grade 6±1<br>G (50.5%); B (49.5%)	**Outcome variable**<br>CIM (territorial range area and distance)<br>**Measurement**<br>ArcGIS (7-day location and activity profiles); Questionnaire	License for CIM and roaming allowance were mediators between perceived walking facilities, crime safety, and neighbourhood relations, and children's territorial range.

		Female (85.7%); Male (14.3%)		**Independent variable(s)**<br>Socio-demographics; Parental perceptions of neighbourhood environment; CIM parenting practices (license for CIM); Neighbourhood Environment walkability scale; Parent-reported children's roaming allowance<br>**Measurement**<br>Questionnaire	
Wolfe et al. 2016<br>United States	Cross-sectional	n=305 *	10-14 years<br>Mean age 12(1.4)<br>G (51%); B (49%)	**Outcome variable**<br>CIM (7 mobility licenses) (0-7 scale)<br>**Measurement**<br>Survey (2006-2007) (Parent)<br>**Independent variable(s)**<br>Parental perceptions of environment<br>Built environment<br>Child & household characteristics<br>**Measurement**<br>Survey (2006-2007) (Parent)	Age is + associated with CIM and Hispanic children experience more restrictions of IM. Parental perceptions of social cohesion and safety are + associated with CIM. Housing unit density was + associated with CIM.

Positive association: +; Negative association: -; Children's independent mobility: CIM; Physical activity: PA; Girl: G; Boy: B; Female: F; Male: M; Time point 1: T1; Time point 2: T2; International physical activity questionnaire: IPAQ; Geographic Information Systems software: GIS; Resilience for Eating and Activity Despite Inequality: READ; Kids in the City: KITC; Environmental Support for Leisure and Active Transport: SALTA; RESIDential Environment Study: RESIDE; Millennium Cohort Study: MCS; Active Transportation Independent Mobility Study: ATIM; Belgian Environmental Physical Activity Study: BEPAS; * indicates from whom data were collected

Table S3. Summary of criteria for quality assessment of included studies

Criteria	Description	N (%)
Objectives	Were the objectives or hypotheses of the research stated?	53 (100)
Study design	Was the study design appropriate for the research undertaken (e.g., cross sectional or longitudinal study, or intervention study with cross sectional analysis)	53 (100)
Target population	Was the target population defined?	53 (100)
Random sample	Was a random sample of the target population taken?	16 (30)
Response rate	Was the response rate 60% or more?	20 (38)
Participant selection	Was the participant selection described?	53 (100)
Participant recruitment	Was the participant recruitment described or referred to?	53 (100)
Participant numbers	Were the numbers of participants at each stage of the study reported? (authors should report at least numbers eligible, numbers recruited, numbers included/excluded from analysis)	52 (98)
Participant inclusion/exclusion	Were criteria for inclusion and/or exclusion of participants in the analysis used?	41 (77)
Study population	Was the study population sufficiently described? (minimum description =sample size, gender, age and an indicator of socioeconomic status)	53 (100)
Variables	Were the measures of IM described?	53 (100)
Data sources	Did the authors describe the source of their data? (e.g. questionnaire, survey, interview, focus group, direct observation, accelerometry, GPS)	53 (100)
Data collection	Did the authors describe how the data were collected? (e.g. by mail., computer, face to face, objective measurement)	53 (100)
Measurement (IM)	Were reliable and valid measures of IM used?	19 (36)
Statistical methods	Were appropriate statistical methods used and described?	53 (100)
Adjustments	Were covariates/confounders controlled for in the analysis? (the authors should present confounder-adjusted estimates)	45 (85)
Missing data	Were the numbers/percentages of participants with missing data for IM reported AND did at least 80% of enrolled participants provide complete data in order to be included in the analysis	23 (43)

Criteria for quality assessment and number (%) of studies scoring pints (either 0.5 or 1) for each criterion.

Table S4. Associations of social-ecological correlates of children's independent mobility

		Related to Children's Independent Mobility			Summary Code		
	**Correlate of IM**	**Studies**	**Association (- / +)**	**Unrelated to Children's Independent Mobility**	**Association**	**Related/ Unrelated**	**% Studies**
**Individual Child Characteristics**							
	Age	Aggio et al. 2017; Alparone et al., 2012; Ayllón et al. 2019; Ayllón et al. 2020 (i, ii) ; Broberg et al. 2013*b*; Buliung et al. 2017; Carver et al. 2012; Cervesato et al. 2019 (i, ii); Chaudhury et al. 2017; Christian et al. 2015 (i,ii), 2016; Cordovil et al., 2015; Delisle Nyström et al. 2019 ; Fyhri et al. 2009; Ghekiere et al. 2017; He et al. 2017; Herrador-Colmenero et al., 2017; Janssen et al. 2016; Johansson, 2006 (i,ii); Kyttä et al. 2015 (i-iii); Lam et al. 2014; Larsen et al. 2015; Love et al. 2020 (T1) ; Mammen et al. 2012; Mitra et al. 2014; Pacilli et al. 2013; Prezza et al. 2001; Scheiner et al. 2019 (i, ii) ; Sharmin et al. 2020 ; Smith et al. 2019 ; Stark et al. 2018; Veitch et al. 2008 (i,ii); Wolfe et al. 2016	+	Ayllón et al. 2020; Broberg et al. 2013*a*; Carver et al. 2013; Cervesato et al. 2019 (i) Herrador-Colmenero et al. 2017; Lin et al. 2017 ; Love et al. 2020 (T2) ; Sharmin et al. 2020	++	42/52	81%
		Carver et al. 2013; Cervesato et al. 2019	-				
	Gender (boy)	Aggio et al., 2017; Alparone et al. 2012; Broberg et al. 2013*a*; Carver et al. 2012, 2013 (Australia), 2014; Christian et al. 2015, 2016; Cordovil et al. 2015 (i,ii); Delisle Nyström et al. 2019 ; Fyhri et al. 2009; Ghekiere et al. 2017; Kyttä, 2004 (Belarus); Lopes et al. 2014 (i,ii); Mitra et al. 2014; Pacilli et al. 2013;	+	Ayllón et al. 2019 ; Ayllón et al. 2020 ; Bhosale et al. 2017 (i,ii); Broberg et al. 2013*b*; R. N. Buliung et al. 2017; Carver et al. 2013; Cervesato et al. 2019; Chaudhury et al. 2017; He et al. 2017; Herrador-Colmenero et al. 2017;	??	27/54	50%

		Prezza et al. 2001; Riazi et al. 2019 ; Schoeppe et al. 2016*a*; Sharmin et al. 2020 ; Smith et al. 2019 ; Villanueva et al. 2014; Wolfe & McDonald, 2016)		Huertas-Delgado et al. 2018; Janssen et al. 2016; Johansson, 2006; Kyttä, 2004 (Finland); Lam et al. 2014; Lin et al. 2017; Lopes et al. 2014 (i,ii); Love et al. 2020 (T2) ; Mammen et al. 2012; Sharmin et al. 2020 ; Stark et al. 2018; Veitch et al. 2008; Wolfe et al. 2016			
	Gender	Veitch et al. 2008; Cervesato et al. 2019	-				
	Ethnicity and/or race	Aggio et al., 2017; Ayllón et al. 2019 Chaudhury et al., 2017; Mammen et al., 2012; Mitra et al., 2014; Riazi et al. 2019 (b ; g); Smith et al. 2019 ; Wolfe et al., 2016	+	He et al., 2017; Janssen et al., 2016; Lin et al., 2017)	++	9/12	75%
	Perceived competence	Ayllón et al. 2020 ; Fyhri & Hjorthol, 2009; Johansson, 2006; Love et al. 2020 (T1) ; Villanueva et al. 2012, 2014	+	Riazi et al. 2019 (i, ii); Love et al. 2020 (T2)	++	6/10	60%
		Riazi et al. 2019 (g)	-				
	Access or ownership of car	Carver et al. 2014 (T2, b); Cervesato et al. 2019 ; Cordovil et al. 2015; Fyhri et al. 2009; He et al. 2017; Johansson, 2006; Kyttä et al. 2015; Lin et al. 2017; Mammen et al. 2012 ; Sharmin et al. 2020	-	Buliung et al. 2017; Carver et al. 2014; Cervesato et al. 2019 ; Delisle Nyström et al. 2019 ; Fyhri et al. 2009; Mammen et al. 2012 ; Riazi et al. 2019 (b; g) ; Sharmin et al. 2020	??	10/20	50%
		He et al., 2017	+				
	Ownership of house or house keys	Ayllón et al. 2019 (i, ii) ; Ayllón et al. 2020 ; Delisle Nyström et al. 2019		Riazi et al. 2019 (b; g)	++	4/6	67%
**Interpersonal Characteristics**							

Parents' Characteristics	Parent age	Ayllón et al. 2019	+	Christian et al. 2015; Ghekiere et al. 2017; Schoeppe et al. 2016*b ;* Riazi et al. 2019 (b ; g)	00	1/6	17%
	Parent gender	Schoeppe et al., 2016*b* (Women); Riazi et al. 2019 (Women; b)	-	Christian et al. 2015; Ghekiere et al. 2017 ; Riazi et al. 2019 (g)	??	2/5	40%
	Parent educational level	Schoeppe et al. 2016*b* (Low)	-	Ayllón et al 2019 ; Christian et al. 2015; Delisle Nyström et al. 2019 ; Fyhri et al. 2009; Janssen et al. 2016 ; Love et al. 2020 (T2) ; Riazi et al. 2019 (b ; g)	00	1/9	11%
	Socioeconomic status (Low)	Aggio et al. 2017; He et al. 2017 (i-iii); Lam et al. 2014 (i,ii); Loo et al. 2015	+	Cervesato et al. 2019 (i, ii); Ghekiere et al. 2017; Janssen et al. 2016; Mammen et al. 2012; Wolfe et al. 2016	??	7/13	54%
	Work status or employment status	Ayllón et al. 2019 (Mother); He et al. 2017 (i-iv); Scheiner et al. 2019 (i, ii)	+	Ayllón et al. 2019 (Father); Fyhri et al. 2009; He et al. 2017 (Father); Mammen et al. 2012; Mitra et al. 2014; Stark et al. 2018 (Father) ; Scheiner et al. 2019 (i, ii); Riazi et al. 2019 (b; g)	??	7/20	37%
		Buliung et al. 2017 (Parent; stay-at-home father)(i,ii); Kyttä et al. 2015	-				
	Parents' perception of child confidence	Ayllón et al. 2019; Ghekiere et al. 2017 (i,ii); Villanueva et al. 2012, 2014; Scheiner et al. (i, ii)	+	Veitch et al. 2017(i,ii)	++	7/10	70%
		Ayllón et al. 2019; Curtis et al., 2015	-				
	Parents' perception of active school travel benefits	Mammen et al. 2012(i, ii)	+	Mammen et al. 2012 (i-iii)	??	2/5	40%

	Parents' attitude toward independent mobility	Ayllón et al. 2019; Alparone et al. 2012; Curtis et al. 2015; Johansson, 2006; Mitra et al. 2014	+		++	5/7	71%
		Ayllón et al. 2019; Curtis et al. 2015	-				
	Encouragement	Carver et al. 2014 (T2, g)	-	Carver et al. 2014 (i,ii); Ghekiere et al. 2017 (i-iii)	00	1/6	17%
Parents' Behaviour	Parents' PA level	Santos et al. 2013	+	Ghekiere et al. 2017; Janssen et al. 2016 (i,ii)	00	1/4	25%
	Parents' policies regarding independent play/travel	Carver et al. 2014 (T1, T2, b)	+	Carver et al. 2014	??	2/5	40%
		Carver et al. 2012 (g); Scheiner et al 2019 (i,ii)	-				
	Travel mode	Riazi et al. 2019 (b)	+	Riazi et al. 2019 (b, i-iii; g, i-iv)	0	1/77	14%
Household characteristics	Household structure	Cervesato et al. 2019	-	Cervesato et al. 2019 ; Janssen et al., 2016; Schoeppe et al., 2016*b*; Scheiner et al. 2019 ; Wolfe et al., 2016 (i,ii)	00	1/7	14%
	Sibling(s)	Ayllón et al. 2019 ; Carver et al. 2014 (T1); Cervesato et al. 2019 (i, ii, iii); Christian et al. 2015 (i,ii), 2016 (i-xi); Johansson, 2006; Lin et al. 2017 ; Scheiner et al. 2019 (i, ii) ; Sharmin et al. 2010 (i, ii)	+	Christian et al. 2015; He & Giuliano, 2017; Janssen et al. 2016; Riazi et al. 2019 (b; g); Wolfe et al. 2016	++	24/31	77%
		Loo et al., 2015	-				
	Birth order	Alparone et al. 2012; Ayllón et al. 2019; Prezza et al. 2001			+	2/3	67%
**Social environment**							
Children's perceptions	Children's positive perceptions of safety	Buliung et al. 2017; Herrador-Colmenero et al. 2017; Love et al. 2020 (T2) ; Villanueva et al. 2012 (g), 2014	+	Huertas-Delgado et al. 2018(i-iv) ; Riazi et al. 2019 (b; g)	??	5/11	45%
	Children's negative perceptions of safety			Buliung et al. 2017; Huertas-Delgado et al.	00	0/15	0%

				2018 (i-vi); Riazi et al. 2019 (b, i-iii, g, i-iii)			
	Interest	Veitch et al., 2017; Villanueva et al., 2012 (b) ; Ayllón et al. 2020	+	Veitch et al., 2017	+	3/4	75%
	Social norms	Veitch et al., 2017; Villanueva et al., 2014(i,ii)(g)	+	Veitch et al., 2017; Villanueva et al., 2012 ; Love et al. 2020 (T2 ; i, ii)	00	3/9	33%
		Curtis et al., 2015; Love et al. 2020 (T1)	-				
Parents' perceptions	Parents' positive perceptions of safety	Carver et al. 2014 (T1); Huertas-Delgado et al. 2018; Janssen et al. 2016; Johansson, 2006; Love et al. 2020 (T1) Mitra et al. 2014; Santos et al. 2013; Villanueva et al. 2012 (b), 2014 (b)	+	Ghekiere et al. 2017; Lin et al. 2017; Love et al. 2020 (T2) (i,ii); Mammen et al. 2012; Prezza et al. 2001; Villanueva et al. 2012	??	9/17	53%
		Huertas-Delgado et al., 2018	-				
	Parents' negative perceptions of safety	Alparone et al. 2012 (Mother);Ayllón et al. 2019 (i,ii) ; Carver et al. 2014 (T1); Christian et al. 2015 (i,ii); Veitch et al. 2017	-	Carver et al. 2014 (T2); Mitra et al. 2014 (i,ii); Riazi et al. (b, i,ii ; g, i,ii) ; Smith et al. 2019 ; Villanueva et al. 2014	??	7/16	44%
	Concern (stranger danger)	Foster et al. 2014 (g); Huertas-Delgado et al. 2018 (i,ii); Love et al. 2020 (T1) ; Mitra et al. 2014 ; Riazi et al. 2019 (g) ;	-	Foster et al. 2014 (b); Huertas-Delgado et al. 2018 (i,ii); Johansson, 2006; Love et al. 2020 (T2) ; Santos et al. 2013	??	6/15	40%
		Fyhri et al. 2009; Huertas-Delgado et al. 2018 (i,ii)	+				
	Concern (crime)	Janssen et al. 2016; Mammen et al. 2012 ; Vlaar et al. 2019; Riazi et al. 2019 (b)	-	Christian et al. 2015 (i-iii); Ghekiere et al. 2017; Huertas-Delgado et al. 2018 (i,ii); Prezza et al. 2001; Riazi et al. 2019 (b) ; Vlaar et al. 2019 (i, ii) ; Wolfe et al. 2016	00	4/14	29%
	Concern (traffic)	Buliung et al. 2017(i,ii); Carver et al. 2014 (T1); Ghekiere et al. 2017 (g); Huertas-Delgado et al. 2018 (i,ii); Johansson, 2006 (i,ii);	-	Carver et al., 2014 (T2); Christian et al., 2015; Huertas-Delgado et al., 2018 (i-iv); Janssen et al.,	--	16/21	76%

		Mammen et al., 2012; Riazi et al. 2019 (b ; g); Scheiner et al. 2019 (i,ii,iii) ; Villanueva et al. 2012; Wolfe et al. 2016		2016 (i,ii); Love et al. 2020 (T1 ;T2) ; Mitra et al., 2014 (i,ii); Prezza et al. 2001 (Mothers); Riazi et al. 2019 (b; g); Smith et al. 2019; Veitch et al., 2017 (i,ii); Vlaar et al. 2019 (i,ii)			
	Social cohesion	Alparone et al. 2012 (Mothers); Love et al. 2020 (T2) ; Prezza et al. 2001; Villanueva et al. 2012 (b); Vlaar et al. 2019 ; Wolfe et al. 2016	+	Lin et al. 2017; Love et al. 2020 (T1; T2, i-iii)	??	6/12	50%
		Love et al. 2020 (T1)	-				
	Informal social control			Carver et al., 2014; Foster et al. 2014; Johansson, 2006; Lin et al. 2017; Prezza et al. 2001; Riazi et al. 2019 (b, g) ; Wolfe et al. 2016	00	0/8	0%
	Social norms	Christian et al. 2015 (i,ii)	-	Ghekiere et al. 2017; Mitra et al. 2014	?	2/4	50%
**Built and physical environment**							
Density	Destination density	Broberg et al. 2013*a*	+	Vlaar et al. 2019 (i,ii); Wolfe et al. 2016 (i-iii)	0	1/6	17%
	Road density	Carver et al. 2014 (T2; g); Scheiner et al. 2019 (i,ii)	-	Buliung et al. 2017; Carver et al. 2014 (i-iv); Larsen et al. 2015; Scheiner et al. 2019 (i,ii) ; Smith et al. 2019 (i,ii) ; Wolfe et al. 2016 (i-iii)	00	3/16	19%
	Population density	Broberg et al., 2013*a*	+	He et al. 2017 (i-iii)	0	1/5	20%
		Larsen et al., 2015	-				
	Housing/residential density	Broberg et al. 2013*a*; Broberg et al., 2013*b*; Scheiner et al. 2019 ; Vlaar et al. 2019 ; Wolfe et al. 2016	+	Scheiner et al. 2019	++	5/8	63%
		Broberg et al. 2013*a*; Broberg et al., 2013*b*	-				

Destinations	Walking and cycling infrastructure	Veitch et al. 2017	+	Ghekiere et al., 2017; Johansson, 2006; Mammen et al. 2012; Veitch et al. 2017 (i-iii)	00	1/8	13%
		Smith et al. 2019	-				
	Green space	Alparone et al. 2012	+	Broberg et al. 2013*a*; Broberg et al. 2013*b*; Veitch et al. 2017 (i, ii); Villanueva et al. 2012 (i,ii)	00	1/7	14%
	Other local destinations (shopping centre, community centre, supermarket, rec centre, retail shop, school, smaller food store)	Villanueva et al. 2012 (i-iv, b)	+	Christian et al. 2015; Ghekiere et al. 2017; Villanueva et al. 2012 (i-iv); Vlaar et al. 2019 (i,ii) ; Wolfe et al. 2016	00	4/13	31%
Design	Type of housing	Broberg et al. 2013*b*; He et al. 2017 (i,ii); Prezza et al. 2001(i,ii)	+	Buliung et al. 2017; Lin et al. 2017 (i-iii)	??	5/10	50%
		He et al., 2017	-				
	Length of residency in one's home	Larsen et al. 2015; Mitra et al., 2014; Prezza et al. 2001	+	Buliung et al. 2017; Lin et al. 2017	++	3/5	60%
	Access to outdoor space/walking/cycling	Carver et al. 2012 (i,ii)	+	Carver et al. 2014	+	2/3	67%
	Walkability	Riazi et al. 2019 (g; 400m); Villanueva et al., 2012, 2014	+	Riazi et al. 2019 (b, 400m, 1600m)	??	3/7	43%
		Carver et al., 2014 (b); Riazi et al 2019 (g; 1600m)	-				
	Aesthetic quality of neighbourhood			Buliung et al. 2017; Ghekiere et al. 2017; Vlaar et al. 2019 (i, ii)	0	0/4	0%
	Urbanization	Kyttä et al. 2015 (suburban); Loo et al. 2015 (suburban); Lopes et al. 2014 (i,ii, highly urbanized) Lopes et al. 2014 (i-v, non-urbanized); Lopes et al. 2014 (i-v, moderately urbanized)	+	Cervesato et al. 2019 (i-iv) ; Cordovil et al. 2015; Delisle Nyström et al. 2019 ; Fyhri et al. 2009; Ghekiere et al. 2017; Kyttä, 2004; Love et al. 2020 (T2); Mammen et al. 2012;	??	14/35	40%

				Riazi et al. 2019 (b ; g, i, ii) Schoeppe et al. 2016*b*			
		Alparone et al., 2012; Cervesato et al. 2019 (i-iv); Lopes et al., 2014 ; Riazi et al. 2019 (b)	-				
Diversity	Land use mix	Carver et al., 2014 (T2, g) ; Sharmin et al. 2019	+	Buliung et al. 2017; Ghekiere et al. 2017 ; Scheiner et al. 2019 ; Sharmin et al. 2019 ; Vlaar et al 2019 (i,ii)	00	2/10	20%
		Scheiner et al. 2019 ; Sharmin et al. 2019	-				
	Socioeconomic status / neighbourhood socioeconomic deprivation (low income)	Love et al. 2020 (T1) ; Mitra et al. 2014; Veitch et al. 2008 (i,ii)	+	Carver et al. 2014; Delisle Nyström et al. 2019; Scheiner et al. 2019	??	4/7	57%
Distance	Distance (short)	Aggio et al. 2017; Buliung et al. 2017; Cervesato et al. 2019 (weekday, i-vi ; weekend, i-iv) i,ii) ; Christian et al. 2015 (i,ii); Cordovil et al. 2015; Fyhri et al., 2009 (i,ii); He et al. 2017; Lin et al. 2017; Loo et al. 2015; Love et al. 2020 (T1)(i,ii) ; Mammen et al. 2012; Prezza et al. 2001 ; Scheiner et al. 2019 (i,ii) ; Villanueva et al. 2012 (b)	+	Larsen et al. 2015; Love et al. 2020 (T2)	++	27/30	90%
		Broberg et al., 2013*b*	-				
	Distance/deviation to mother's work	He et al., 2017 (i,ii)	+	He et al., 2017	+	2/3	67%
	Distance/deviation to father's work	He et al., 2017	-	He et al., 2017 (i,ii)	0	1/3	33%
	Proximity to green space	Christian et al., 2015 (i,ii); Prezza et al., 2001; Villanueva et al., 2012 (b)	+		+	4/4	100%

IM=independent mobility; PA=physical activity. Roman numerals indicated number of correlates examined in study. Girl = g; Boy = b; Meters = m; Time point 1: T1; Time point 2: T2; + = positive association (both variables increase); - = negative association (one variable increases while the other decreases); 0 = no association; Based on the percentage of studies supporting the association, the associations were labeled 0-33% → no association (0); 34-59% →

indeterminate or inconsistent (?); 60-100% → positive or negative association (+ or -). If more than 4 studies supported the association in the same direction, it was labeled 00, ??, ++, or --. The ?? code indicated that a variable has been frequently studied with considerable lack of consistency in findings. Broberg et al., 2013*a* = Broberg, Kyttä, & Fagerholm: Child-friendly urban structures: Bullerby revisited; Broberg 2013*b* = Broberg, Salminen, & Kyttä: Physical environmental characteristics promoting independent and active transport to children's meaningful places; Schoeppe 2015*a* = Schoeppe, Tranter, Duncan, Curtis, Carver, & Malone: Australian children's independent mobility levels: secondary analyses of cross-sectional data between 1991 and 2012; Schoeppe 2015*b* = Schoeppe, Duncan, Badland, Rebar, & Vandelanotte: Too far from home? Adult attitudes on children's independent mobility range.

Table S5. Sample age ranges of included studies (n=53)

<table>
<tr><th>Age (Years)</th><th>5</th><th>6</th><th>7</th><th>8</th><th>9</th><th>10</th><th>11</th><th>12</th><th>13</th><th>14</th><th>15</th><th>16</th><th>17</th><th>18</th></tr>
<tr><th>Grade Level</th><th></th><th>1</th><th>2</th><th>3</th><th>4</th><th>5</th><th>6</th><th>7</th><th>8</th><th>9</th><th>10</th><th>11</th><th>12</th><th></th></tr>
<tr><td></td><td></td><td></td><td></td><td colspan="3">Alparone & Pacilli, 2012</td><td></td><td></td><td></td><td></td><td></td><td></td><td></td><td></td></tr>
<tr><td></td><td></td><td></td><td></td><td></td><td></td><td colspan="6">Broberg, Kyttä, et al., 2013</td><td></td><td></td><td></td></tr>
<tr><td></td><td></td><td></td><td></td><td></td><td></td><td></td><td>Broberg, Salminen, et al., 2013</td><td></td><td></td><td>Broberg, Salminen, et al., 2013</td><td></td><td></td><td></td><td></td></tr>
<tr><td></td><td></td><td></td><td></td><td></td><td colspan="6">Carver et al., 2012</td><td></td><td></td><td></td><td></td></tr>
<tr><td></td><td></td><td></td><td></td><td></td><td></td><td colspan="3">Carver et al., 2013</td><td></td><td></td><td></td><td></td><td></td><td></td></tr>
<tr><td></td><td></td><td></td><td></td><td></td><td colspan="3">Carver et al., 2014</td><td></td><td></td><td></td><td></td><td></td><td></td><td></td></tr>
<tr><td></td><td></td><td></td><td></td><td></td><td colspan="4">Chaudhury et al., 2017</td><td></td><td></td><td></td><td></td><td></td><td></td></tr>
<tr><td></td><td></td><td></td><td></td><td></td><td></td><td colspan="3">Christian et al., 2014</td><td></td><td></td><td></td><td></td><td></td><td></td></tr>
<tr><td></td><td></td><td></td><td></td><td colspan="8">Christian et al., 2015</td><td></td><td></td><td></td></tr>
<tr><td></td><td></td><td></td><td></td><td colspan="8">Christian et al., 2016</td><td></td><td></td><td></td></tr>
<tr><td></td><td></td><td></td><td></td><td colspan="8">Cordovil et al., 2015</td><td></td><td></td><td></td></tr>
<tr><td></td><td></td><td></td><td></td><td></td><td></td><td colspan="3">Foster et al., 2014</td><td></td><td></td><td></td><td></td><td></td><td></td></tr>
<tr><td></td><td></td><td colspan="7">Fyhri &Hjorthol, 2009</td><td></td><td></td><td></td><td></td><td></td><td></td></tr>
<tr><td></td><td></td><td></td><td></td><td></td><td></td><td colspan="3">Ghekiere et al., 2017</td><td></td><td></td><td></td><td></td><td></td><td></td></tr>
<tr><td></td><td colspan="14">He & Giuliano, 2017</td></tr>
<tr><td></td><td></td><td></td><td colspan="6">Johansson, 2006</td><td></td><td></td><td></td><td></td><td></td><td></td></tr>
<tr><td></td><td></td><td></td><td></td><td colspan="2">Kyttä, 2004</td><td></td><td></td><td></td><td></td><td></td><td></td><td></td><td></td><td></td></tr>
<tr><td></td><td></td><td></td><td colspan="9">Kyttä et al., 2015 (also 8-10)</td><td></td><td></td><td></td></tr>
<tr><td></td><td></td><td colspan="7">Lam & Loo, 2014</td><td></td><td></td><td></td><td></td><td></td><td></td></tr>
<tr><td></td><td></td><td colspan="7">Larsen et al., 2015</td><td></td><td></td><td></td><td></td><td></td><td></td></tr>
<tr><td></td><td></td><td></td><td></td><td colspan="6">Lin et al., 2017</td><td></td><td></td><td></td><td></td><td></td></tr>
<tr><td></td><td></td><td colspan="7">Loo & Lam, 2015</td><td></td><td></td><td></td><td></td><td></td><td></td></tr>
<tr><td></td><td></td><td></td><td></td><td colspan="8">Lopes et al., 2014</td><td></td><td></td><td></td></tr>
<tr><td></td><td></td><td colspan="9">Mammen et al., 2012</td><td></td><td></td><td></td><td></td></tr>
</table>

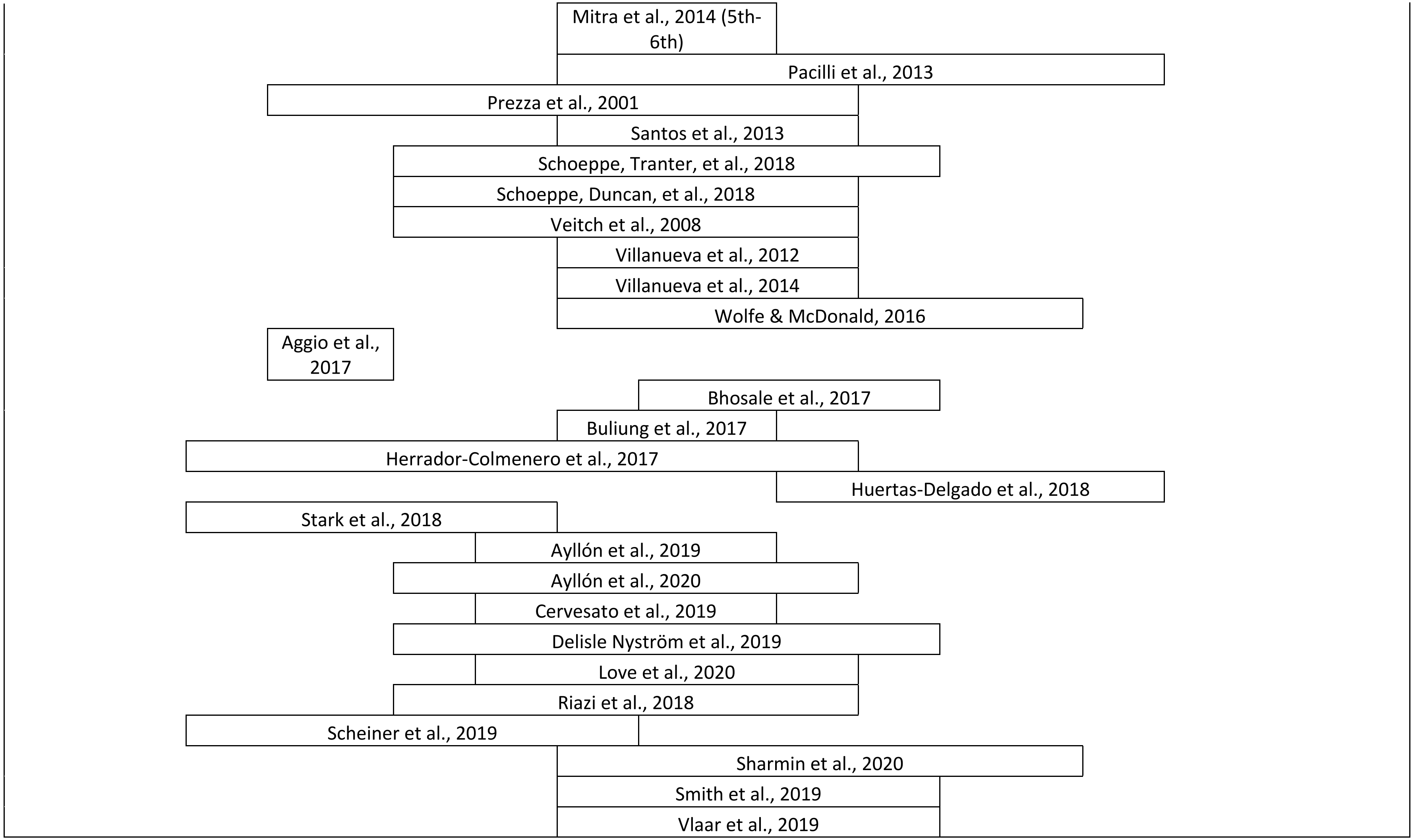
Mitra et al., 2014 (5th-6th)
Pacilli et al., 2013
Prezza et al., 2001
Santos et al., 2013
Schoeppe, Tranter, et al., 2018
Schoeppe, Duncan, et al., 2018
Veitch et al., 2008
Villanueva et al., 2012
Villanueva et al., 2014
Wolfe & McDonald, 2016
Aggio et al., 2017
Bhosale et al., 2017
Buliung et al., 2017
Herrador-Colmenero et al., 2017
Huertas-Delgado et al., 2018
Stark et al., 2018
Ayllón et al., 2019
Ayllón et al., 2020
Cervesato et al., 2019
Delisle Nyström et al., 2019
Love et al., 2020
Riazi et al., 2018
Scheiner et al., 2019
Sharmin et al., 2020
Smith et al., 2019
Vlaar et al., 2019
